# Fluoroquinolone resistance in ESKAPE pathogens: evolutionary pathways, one health transmission, and clinical surveillance

**DOI:** 10.3389/fmicb.2025.1719066

**Published:** 2026-01-13

**Authors:** Ayman Elbehiry, Eman Marzouk, Adil Abalkhail

**Affiliations:** Department of Public Health, College of Applied Medical Sciences, Qassim University, Buraydah, Saudi Arabia

**Keywords:** efflux pumps, ESKAPE pathogens, fluoroquinolone resistance, plasmid-mediated quinolone resistance, public health, quinolone resistance determiningregion mutations

## Abstract

Fluoroquinolones (FQs) remain important treatments for many Gram-negative and some Gram-positive infections, but rapid resistance development is steadily reducing their clinical usefulness. This review integrates biological and epidemiologic evidence through a One Health perspective focused on the ESKAPE group: *Enterococcus faecium*, *Staphylococcus aureus*, *Klebsiella pneumoniae*, *Acinetobacter baumannii*, *Pseudomonas aeruginosa*, and *Enterobacter* spp. At the molecular level, resistance often begins with changes in quinolone-resistance determining regions of DNA gyrase and topoisomerase IV, followed by spread through plasmid-mediated mechanisms including *qnr*, *aac(6′)-Ib-cr*, *qepA*, and *oqxAB*. Species-specific efflux pumps such as NorA, AcrAB–TolC, and OqxAB, along with outer membrane and porin alterations, further contribute to resistance. Co-selection on mobile elements, including IncX, IncF, and IncL plasmids that may also carry ESBL or AmpC genes, enhances dissemination. Extrapatient reservoirs, including external hospitals, veterinary medicine, food-animal production, and contaminated water, sustain selection pressure and support horizontal transmission. Rising minimum inhibitory concentrations (MICs) are diminishing the reliability of empiric FQ therapy. Pharmacokinetics and pharmacodynamics are central to this trend; suboptimal exposure, such as ciprofloxacin AUC/MIC below 125 in Gram-negative infections, increases the time within the mutant-selection window and favors first-step mutants. Mechanism-based strategies include target-attaining dosing, early optimization of therapy, use of combinations that address efflux or permeability barriers, and stewardship guided by local MIC distributions. Emerging priorities include AI-based prediction of resistance trajectories, efflux and plasmid-transfer inhibitors, and phage or nanoparticle systems designed to reduce pathogen burden, disrupt biofilms, generate reactive oxygen species, or deliver site-directed therapy. Integration of rapid diagnostics will support these efforts and help preserve FQ effectiveness.

## 1 Introduction

Fluoroquinolones (FQs) remain central to infectious-disease practice because they combine broad Gram-negative activity, convenient Gram-positive coverage, favorable oral bioavailability, and deep tissue penetration (Dalhoff, 2012). Cross-pharmacokinetic summaries and agent-specific analyses confirm these advantages and support their continued use in routine care (Turnidge, 1999; Pham et al., 2019). However, widespread use in both community and hospital settings increases selection pressure and accelerates the emergence and spread of resistance (World Health Organization, 2022a; Ajulo and Awosile, 2024).

This selective pressure is particularly consequential among the ESKAPE pathogens *Enterococcus faecium (E. faecium)*, *Staphylococcus aureus* (*S. aureus*), *Klebsiella pneumoniae* (*K. pneumoniae*), *Acinetobacter baumannii* (*A. baumannii*), *Pseudomonas aeruginosa (P. aeruginosa)*, and *Enterobacter* spp., which dominate contemporary antimicrobial resistance (AMR) burdens owing to their combined virulence, ecological adaptability particularly to healthcare environments and their capacity to evade multiple antibiotic classes (Miller and Arias, 2024).

At the same time, the World Health Organization (WHO) has established the Global Antimicrobial Resistance and Use Surveillance System (GLASS), which standardizes reporting and links AMR with antimicrobial use (AMU) through a One Health model (Tornimbene et al., 2022; World Health Organization, 2022b). Recent analyses emphasize that integrating AMU and resistance metrics across human, animal, food, and environmental sectors is essential for accurately interpreting resistance trajectories among ESKAPE pathogens within a One Health framework (Ajulo and Awosile, 2024; Oyenuga et al., 2024).

Evidence from food systems further reinforces this One Health perspective, as AMR ESKAPE pathogens are increasingly detected in animal-derived products intended for human consumption. Studies of dairy products, for example, have documented the presence of multidrug-resistant *S. aureus*, *E. coli*, and *P. aeruginosa*, highlighting how agricultural practices and food-processing environments can act as reservoirs and transmission interfaces for clinically relevant resistance determinants (Nadi et al., 2024).

At the molecular level, resistance usually begins with sequential changes in the quinolone-resistance determining regions (QRDRs) of DNA gyrase and topoisomerase IV, which raise the minimum inhibitory concentration (MIC) and set the stage for further evolution (Hooper and Jacoby, 2015; Shaheen et al., 2021). Structural studies show that quinolones stabilize gyrase or topoisomerase IV bound to DNA and that classic QRDR substitutions such as *gyrA* S83L or D87N and *parC* S80I or E84V weaken drug–target binding and increase MICs (Aldred et al., 2014). These conserved mutational pathways represent a shared evolutionary entry point for fluoroquinolone resistance (FQ-R) across ESKAPE pathogens, reflecting convergent adaptation to quinolone selection pressure (Miller and Arias, 2024; Venkateswaran et al., 2023).

These chromosomal modifications are often accompanied by plasmid-mediated quinolone resistance (PMQR) determinants, including *qnr* families, *aac*(6′)-Ib-cr, *qepA*, and *oqxAB*, which reduce intracellular drug exposure and facilitate the fixation of subsequent QRDR mutations (Yanat et al., 2017; Gomi and Adachi, 2025). PMQR plasmids commonly carry plasmid-mediated AmpC or extended-spectrum *β*-lactamase (ESBL) genes on IncX, IncF, or IncL backbones, supporting co-selection and dissemination in clinical and environmental reservoirs (Yanat et al., 2017; Gomi and Adachi, 2025). One Health investigations increasingly document these plasmid architectures across livestock, food products, and environmental reservoirs, underscoring the role of agricultural and food-chain interfaces in sustaining PMQR–ESBL co-circulation beyond clinical settings (Oyenuga et al., 2024; Khasapane et al., 2024; Kumar et al., 2025).

These early genetic events are closely tied to pharmacokinetics and pharmacodynamics (PK/PD) (Van et al., 2019). The mutant-selection window (MSW) hypothesis proposes that first-step mutants are enriched when drug concentrations fall between the wild-type MIC and the mutant-prevention concentration (MPC), guiding antimutant dosing strategies (Drlica, 2003; Drlica and Zhao, 2007). Evidence continues to support MSW and MPC concepts as tools to limit early mutant amplification (Drlica and Zhao, 2007; Krajewska et al., 2023). For ciprofloxacin, an AUC/MIC ratio of at least 125 is associated with bactericidal activity and reduced selection pressure, yet rising MICs and standard dosing often limit target attainment and highlight the need for dose optimization (Haeseker et al., 2013; De Vroom et al., 2020). Surveillance data from human, veterinary, and environmental settings indicate that increasing baseline MIC distributions among ESKAPE pathogens further constrain PK/PD target attainment (Khasapane et al., 2024; Koutsoumanis et al., 2021).

From an epidemiologic perspective, FQ-R has expanded through high-risk clones and mobile elements that circulate across health systems and national borders (Sati et al., 2025). In *E. coli*, sequence type 131 (ST131), particularly H30 and H30Rx, is strongly associated with FQ-R, ESBL carriage, and global dissemination (Price et al., 2013; Johnson et al., 2017; Lindblom et al., 2022). In *K. pneumoniae*, FQ-R frequently accompanies ESBL and carbapenemase plasmids, while porin loss and efflux activation further increase multidrug resistance (MDR) (Lascols et al., 2023; Li et al., 2023; Li et al., 2025).

In *S. aureus*, NorA overexpression and topoisomerase IV-driven mutational pathways shift isolates from reduced susceptibility to high-level resistance, and NorA-targeted potentiators remain under investigation (Yu et al., 2022; Aeschlimann et al., 1999; Truong-Bolduc et al., 2024; Shobana et al., 2024). In *A. baumannii*, constitutive AdeIJK contributes to intrinsic nonsusceptibility, while adeRS-mediated upregulation of AdeABC supports acquired resistance and persistence in intensive-care units (ICUs) (Yoon et al., 2015; Xu et al., 2019; Darby et al., 2023; Xie et al., 2025; Wimalasekara et al., 2025).

In *P. aeruginosa*, QRDR mutations act synergistically with resistance-nodulation-division efflux systems, particularly MexAB-OprM, producing marked MIC increases (Nakajima et al., 2002; Bruchmann et al., 2013; Cabrera et al., 2022). In *Enterobacter* spp., resistance evolves stepwise through QRDR mutations combined with reduced permeability and enhanced efflux (Guillard et al., 2015). In *E. faecium*, high-level quinolone resistance is linked to combined *gyrA* and *parC* mutations within broader composite resistance profiles (El Amin et al., 1999; Amuasi et al., 2023). Data from outpatient clinics, Data from outpatient settings,

One Health surveillance, and GLASS reports collectively demonstrate selection and transmission of quinolone resistance across human, animal, and environmental reservoirs (Ajulo and Awosile, 2024; Tornimbene et al., 2022). These species-specific pathways extend beyond hospitals into livestock, food systems, and environmental reservoirs, reinforcing the need for coordinated One Health stewardship and surveillance (Oyenuga et al., 2024; Venkateswaran et al., 2023; Khasapane et al., 2024).

**Figure 1:**
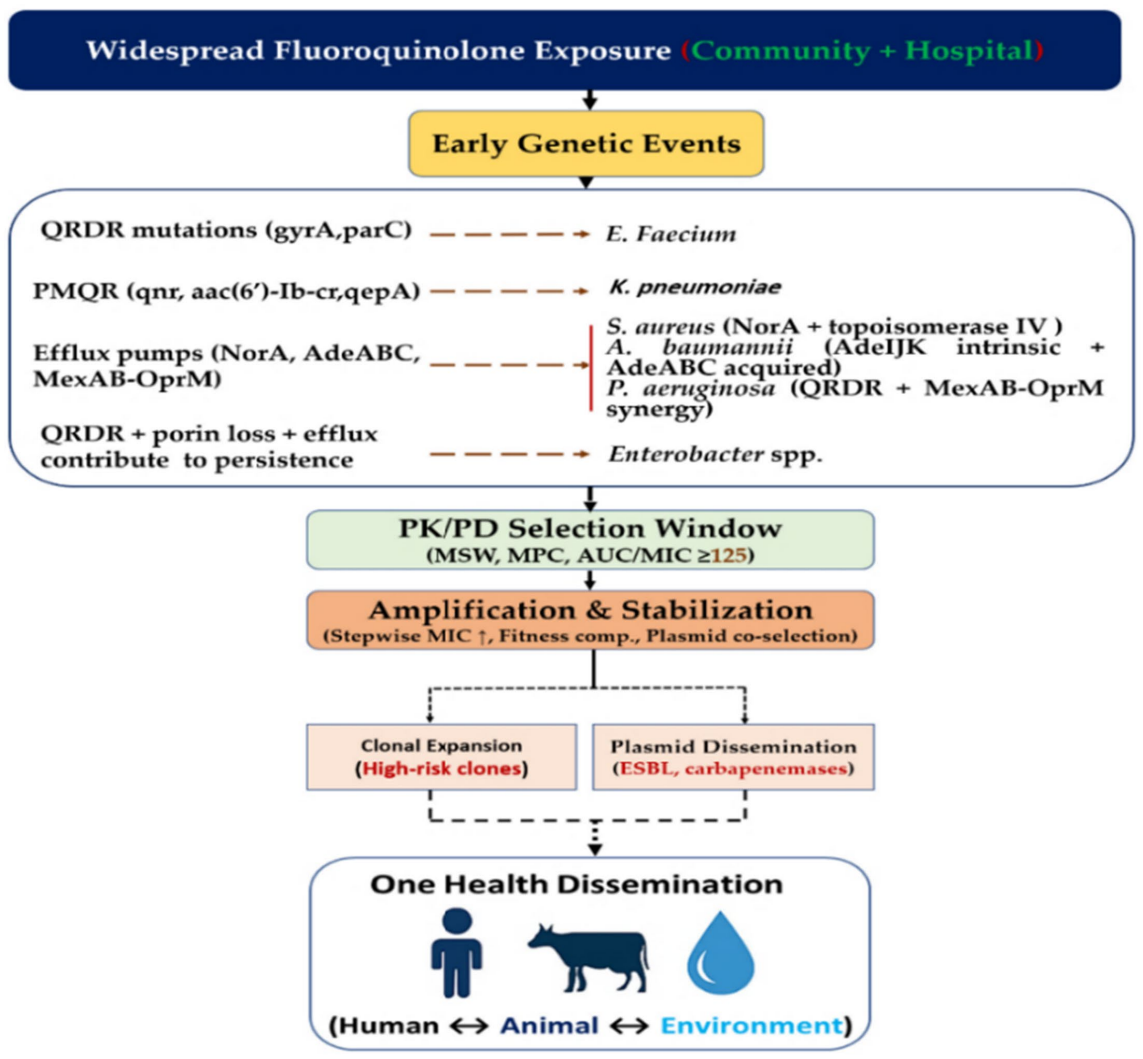
Evolutionary roadmap of FQ-R in the ESKAPE pathogens. Resistance begins with QRDR changes in *gyrA/gyrB* and *parC/parE*, followed by acquisition of PMQR genes (*qnr*, *aac(6′)-Ib-cr*, *qepA*, *oqxAB*), efflux upregulation, and porin loss in selected species. Within PK/PD limits defined by the MSW, MPC, and AUC/MIC, these factors determine suppression or amplification of resistant subpopulations. The figure also outlines downstream effects, including rising MICs, clonal expansion, plasmid dissemination, and One Health transmission. Species-specific amplifiers such as NorA, MexAB-OprM, AdeABC/AdeIJK, OqxAB/AcrAB–TolC, and ST131 are indicated.

Figure 1 summarizes this progression. Resistance typically begins with QRDR mutations that are reinforced by PMQR determinants such as *qnr*, *aac*(6′)-*Ib*-*cr*, *qepA*, and *oqxAB*. These changes are followed by efflux overexpression and porin loss, all constrained by PK and PD parameters, including the MSW, MPC, and AUC/MIC. These steps link molecular evolution with rising MICs, clonal expansion, plasmid dissemination, and cross-sector transmission.

We performed a narrative search covering January 1999 to September 2025 in PubMed/MEDLINE, Embase, Scopus, and Web of Science. Search terms included fluoroquinolones, QRDR, PMQR, *qnr*, *aac*(6′)-*Ib*-*cr*, *qepA*, *oqxAB*, efflux systems (NorA, AcrAB–TolC, and RND pumps), outer membrane and porins, AUC/MIC, mutant-selection window, mutant-prevention concentration, GLASS, and One Health. We prioritized original studies, meta-analyses, and authoritative surveillance reports. Non-English and non-peer-reviewed sources were excluded, except for global AMR guidance documents.

From these sources, we synthesized early genetic resistance events and their pharmacokinetic and pharmacodynamic context. We compared species-specific resistance pathways across the six ESKAPE pathogens, with emphasis on efflux regulation, compensatory mechanisms, and plasmid ecology. These pathways were then integrated with evidence on clonal dissemination and plasmid-mediated transmission within a One Health framework.

In summary, this review links molecular resistance mechanisms, from QRDR mutations to PMQR determinants, efflux activation, and porin modification, with PK and PD selection processes to define an ESKAPE-wide evolutionary framework for FQ. By embedding these mechanisms within a One Health perspective, the review aligns molecular resistance pathways with cross-sector transmission patterns reported in recent global AMR analyses.

## 2 Historical milestones of FQ-R

### 2.1 Discovery and clinical adoption of quinolones/FQs

The development of quinolones began unexpectedly when a chloroquine byproduct showed antibacterial activity, which prompted the synthesis and commercialization of nalidixic acid (Bisacchi, 2015). Clinical use started in 1967, mainly for urinary tract infections (UTIs) (Emmerson and Jones, 2003). To improve the narrow spectrum and limited tissue penetration of the early compound, chemists added a fluorine at position C-6. This change enhanced cell entry, tissue distribution, and overall activity (Pham et al., 2019; Rodrigues and Silva, 2025). It led to the first FQs, such as flumequine, and later to human-use agents in the 1980s, including norfloxacin and ciprofloxacin, which expanded the spectrum and supported systemic therapy (Pallo-Zimmerman et al., 2010). Additional side-chain modifications improved penetration, prolonged half-life, and strengthened Gram-positive activity (Emami et al., 2005). Combined with high oral bioavailability, these advantages supported widespread use in both community and hospital settings (Emmerson and Jones, 2003; Appelbaum and Hunter, 2000).

### 2.2 Timeline of first resistance reports among ESKAPE pathogens

Early clinical and laboratory studies identified QRDR changes in DNA gyrase and topoisomerase IV as the initial drivers of resistance, with common substitutions in *gyrA* (S83L/D87N) and *parC* (S80I/E84V) across Enterobacterales and related groups (Tewawong et al., 2025; Mirzaii et al., 2018). In *E. faecium*, most high-level resistant isolates carried combined *gyrA* and *parC* mutations, while a low-level resistant isolate without these changes likely relied on efflux or modifications in other topoisomerase subunits (el Amin et al., 1999). In *P. aeruginosa*, experimental work showed strong synergy between a single *gyrA* mutation and MexAB-OprM derepression, producing a 128 to 1,024 fold rise in MIC, far greater than either mechanism alone (Nakajima et al., 2002). In *Enterobacter* spp., clinical and molecular data identified QRDR mutations combined with reduced permeability and increased efflux as key contributors to stepwise nonsusceptibility during FQ exposure (Guillard et al., 2015).

PMQR was first recognized in 1998, when *qnrA* was detected in a *K. pneumoniae* isolate in the United States. Subsequent reports documented the spread of *qnrA*, *qnrB*, and *qnrS*, followed by *aac(6′)-Ib-cr* and *qepA*, often on plasmids that also carried beta-lactam resistance genes (Poirel et al., 2005; Chen et al., 2006; Peymani et al., 2015). Table 1 summarizes major milestones from nalidixic acid to the discovery of the earliest PMQR determinants.

**Table 1:** Key milestones in quinolone/FQ discovery, adoption, and early resistance signals.

Milestone	Year/Period	Brief note	Reference(s)
Discovery of nalidixic acid	1962	Identified during chloroquine-related synthesis	Bisacchi (2015)
Clinical introduction of nalidixic acid	1967	Early use for UTIs	Emmerson and Jones (2003)
Fluorination at C-6 leading to early FQs	1970s	Improved potency and tissue penetration	Pham et al. (2019) and Rodrigues and Silva (2025)
First systemic FQs (norfloxacin, ciprofloxacin)	1980s	Broader activity and improved PK	Pallo-Zimmerman et al. (2010)
Later generational optimizations	1990s–2000s	Longer half-life and expanded Gram-positive activity	Emami et al. (2005)
Early QRDR substitutions (*gyrA* S83L/D87N; *parC* S80I/E84V)	1990s–2000s	Core mechanism across Enterobacterales and other taxa	Tewawong et al. (2025) and Mirzaii et al. (2018)
*E. faecium* combined *gyrA/parC* mutations	Late 1990s	Present in most high-level resistant strains	El Amin et al. (1999)
*P. aeruginosa gyrA* plus MexAB-OprM synergy	2002	Synergy producing large MIC increases	Nakajima et al. (2002)
*Enterobacter* spp. QRDR plus permeability and efflux changes	2015	Stepwise rise in nonsusceptibility	Guillard et al. (2015)
First PMQR determinant (*qnrA*) in *K. pneumoniae*	1998	Followed by *qnrA/B/S*, *aac(6′)-Ib-cr*, *qepA*	Poirel et al. (2005), Chen et al. (2006), and Peymani et al. (2015)

### 2.3 Early clonal outbreaks that reshaped global epidemiology

Table 2 outlines early outbreaks that shaped global FQ-R, most notably the rise of *E. coli* ST131 and high-risk *P. aeruginosa* lineages. In *E. coli*, the H30 and H30Rx subclades drove widespread extraintestinal infections. Genomic and clinical studies show that FQ-R aligns closely with CTX-M ESBLs, especially *blaCTX-M-15*, which supports the global success of these sublineages (Price et al., 2013; Bevan et al., 2017). Two major branches have since emerged. The C2/H30Rx group carrying *blaCTX-M-15* spread internationally, while C1/H30R strains with *blaCTX-M-27* expanded across several regions, including Japan. Recent reports describe continued dissemination and local establishment of C1-M27 (Matsumura et al., 2016; Tóth et al., 2024; Wranne et al., 2024).

**Table 2:** Early clonal outbreaks that reshaped global epidemiology.

Pathogen/clone	Hallmark features	Epidemiologic impact	Reference(s)
*E. coli* ST131 H30/H30Rx (C2/H30Rx)	FQ-R + ESBL (often *blaCTX-M-15*)	Dominant cause of extraintestinal infections across regions	Price et al. (2013) and Bevan et al. (2017)
*E. coli* C1/H30R (C1-M27)	FQ-R + *blaCTX-M-27*	International emergence (e.g., Japan and beyond) and local establishment	Matsumura et al. (2016), Tóth et al. (2024), and Wranne et al. (2024)
*P. aeruginosa* (multiple hospital lineages)	Chromosomal mechanisms: RND efflux (MexAB-OprM, MexCD-OprJ, MexEF-OprN, MexXY-OprM) + target mutations	Difficult-to-treat nosocomial infections; adaptive resistance	Jeannot et al. (2008) and Morita et al. (2012)

In *P. aeruginosa*, reviews emphasize that chromosomal mechanisms dominate early resistance. RND efflux systems such as MexAB-OprM, MexCD-OprJ, MexEF-OprN, and MexXY-OprM, together with target mutations, generate difficult-to-treat phenotypes in hospitals and chronic infections (Jeannot et al., 2008; Morita et al., 2012). These traits reflect strong intrinsic tolerance and rapid adaptive responses under antibiotic pressure.

## 3 Molecular foundations of resistance

**Figure 2:**
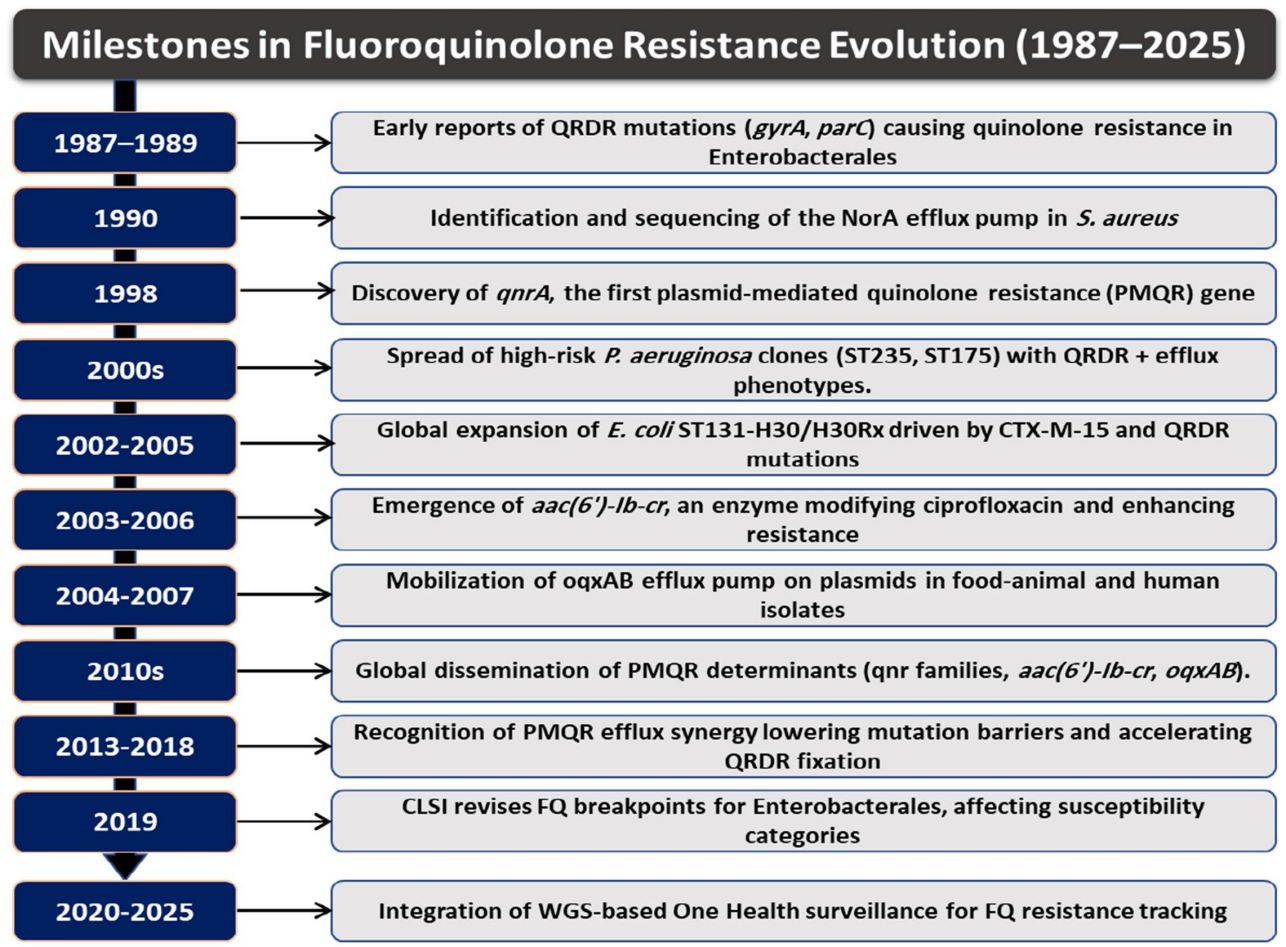
Timeline of key milestones in FQ-R (1987–2025). Major events that shaped the emergence and spread of resistance, including the first reports of QRDR substitutions, identification of efflux systems, appearance of plasmid-mediated quinolone resistance (PMQR), expansion of high-risk lineages, revisions to clinical breakpoints, and incorporation of whole-genome-based One Health surveillance.

Recent narrative syntheses emphasize that AMR in ESKAPE pathogens reflects the convergence of multiple resistance mechanisms rather than isolated pathways, underscoring the need for integrated mechanistic frameworks to understand resistance development and its clinical impact (Shah et al., 2024). The historical milestones, clonal shifts, and surveillance developments summarized in Figure 2 provide the background for the molecular mechanisms discussed in this section. These events set the stage for how the ESKAPE pathogens acquired and stabilized FQ-R through target changes, plasmid exchange, efflux activation, and fitness compensation.

### 3.1 QRDR mutations

Early FQ-R in the ESKAPE group usually begins with substitutions in the QRDRs of *gyrA*, *gyrB*, *parC*, and *parE*. In Gram-negatives, the typical pattern includes *gyrA* S83L or D87N and *parC* S80I or E84V, although species-level variations are common (Hooper and Jacoby, 2015). Double *gyrA* and *parC* mutants occur frequently in ciprofloxacin and nalidixic acid resistant Enterobacterales and often involve the same hotspot residues (Hooper and Jacoby, 2015). In *E. faecium*, high-level resistance was reported in isolates carrying combined *gyrA* and *parC* changes, while a low-level isolate without these substitutions likely depended on efflux or other topoisomerase alterations (Rozen et al., 2007).

Experimental reconstructions in *P. aeruginosa* show strong synergy between a single *gyrA* mutation and derepressed *mexAB-oprM*. The combined mutant increased MICs by about 1,024-fold relative to strains lacking MexAB OprM. Single *gyrA* or *mexR* changes produced smaller effects, and a *gyrA* mutation with wild-type MexAB OprM resulted in intermediate increases (Morita et al., 2012). In *Enterobacter* spp., QRDR substitutions act with reduced permeability and increased efflux to produce gradual nonsusceptibility during exposure (Damier-Piolle et al., 2008).

Across species, target preference reflects cell wall class. Most Gram-negatives acquire *gyrA* changes before *parC*, whereas several Gram-positive pathogens favor topoisomerase IV as the initial target (Hooper and Jacoby, 2015). Comparative analysis also reveals that nonfermenters such as *P. aeruginosa* and *A. baumannii* rely heavily on early efflux upregulation, with QRDR mutations mainly amplifying resistance rather than initiating it (Darby et al., 2023; Rezaei et al., 2024). This explains why identical *gyrA* mutations generate far higher MICs in *P. aeruginosa*, where RND pumps such as MexAB OprM provide strong synergy, compared with Enterobacterales, which have lower baseline efflux activity (Nakajima et al., 2002). In contrast, *S. aureus* and *E. faecium* show topoisomerase IV first trajectories that differ from the gyrase first pattern common in Gram-negative species (Fournier and Hooper, 1998; Oizumi et al., 2001).

These species specific routes, combined with lineage effects such as ST131 in *E. coli* and IC2 or ST2 in *A. baumannii*, show that FQ-R evolves through distinct combinations of efflux, target hierarchy, and clonal background (Darby et al., 2023; Nicolas-Chanoine et al., 2014). This framework reduces descriptive redundancy and links molecular variation to surveillance and stewardship needs.

To minimize redundancy in subsequent sections, the mechanistic descriptions of QRDR mutations and efflux activation provided here form the central reference for species-specific elaboration in Section 4, which therefore focuses on evolutionary and epidemiologic context rather than repeating molecular detail.

### 3.2 Plasmid-mediated quinolone resistance

PMQR consists of three modules that act additively with QRDR mutations. The first includes Qnr proteins encoded by *qnrA*, *qnrB*, *qnrS*, *qnrC*, and *qnrD*. The second is the modifying enzyme *aac(6′)-Ib-cr*, which derives from a common aminoglycoside acetyltransferase and reduces ciprofloxacin activity through N-acetylation (Strahilevitz et al., 2009; Robicsek et al., 2006a). The third includes the efflux determinants *qepA* and *oqxAB*. *qepA* was first identified on plasmid pHPA in an *E. coli* clinical isolate, where transformants showed increased resistance to norfloxacin, ciprofloxacin, and enrofloxacin (Yamane et al., 2007). *oqxAB* is a plasmid-mediated RND pump reported in Enterobacterales and is associated with resistance to nitrofurantoin and quinolones (Ho et al., 2016; Wong et al., 2015; Jacoby et al., 2015). It originated on the IncX1 plasmid pOLA52 in porcine *E. coli*, where it mediates olaquindox resistance and is mobilizable (Norman et al., 2008; Yuan et al., 2012). Co-selection is common, as PMQR genes often occur with ESBLs, carbapenemases, and aminoglycoside resistance on conjugative plasmids, and co-transfer of *qnr* with ESBL genes and *aac(6′)-Ib-cr* has been documented (Strahilevitz et al., 2009; Karah et al., 2010).

### 3.3 Efflux pump systems

Efflux is a central mechanism of FQ-R and varies markedly across ESKAPE pathogens. In *A. baumannii*, AdeIJK confers intrinsic nonsusceptibility, and AdeABC overexpression often driven by *adeRS* supports MDR; both pumps export FQs among other substrates (Xu et al., 2019; Damier-Piolle et al., 2008; Coyne et al., 2011; Yoon et al., 2013). In *P. aeruginosa*, RND systems MexAB–OprM, MexCD–OprJ, MexEF–OprN, and MexXY–OprM mediate FQ efflux. Structural and genetic studies show channel interchangeability, such as MexXY operating with OprM or OprA in PA7, and link pump overexpression with clinical MDR (Morita et al., 2012). In Enterobacterales, chromosomal AcrAB–TolC and plasmid-mediated OqxAB contribute to efflux under global regulation (Jacoby et al., 2015).

In *S. aureus*, NorA overexpression increases quinolone MICs and acts additively with topoisomerase mutations (Hooper and Jacoby, 2015). In Enterobacterales, global regulators marA, soxS, and ramA activate efflux, such as AcrAB–TolC, and suppress porins, whereas marR, soxR, and ramR act as local repressors; environmental cues can shift this balance (Holden and Webber, 2020; Holden et al., 2023). In *A. baumannii*, *adeRS* activates AdeABC, and *adeRS* mutations are linked to AdeABC overproduction and MDR (Xu et al., 2019; Coyne et al., 2011). Table 3 summarizes QRDR hotspots, PMQR determinants, primary efflux systems, their regulators, and common co-selection patterns across the ESKAPE group.

**Table 3:** Core molecular modules of FQ-R in ESKAPE pathogens.

Mechanism	Key determinants	Species highlights	Notes
QRDR mutations	*gyrA* (S83, D87), *parC* (S80, E84); also, *gyrB*, *parE*	Order of target preference varies by species: *E. coli*, *K. pneumoniae*, *Enterobacter* spp. (*gyrA* then *parC*); *S. aureus*, *E. faecium* (*parC* then *gyrA*); *P. aeruginosa* (gyrA mutation combined with MexAB-OprM activity)	Stepwise MIC increases; double or triple substitutions common
PMQR	*qnrA/B/S/C/D*, *aac(6′)-Ib-cr*, *qepA*, *oqxAB*	Most frequent in *K. pneumoniae* and *E. coli*; *oqxAB* originates from IncX1 plasmid pOLA52	Often co-located with ESBLs, carbapenemases, aminoglycoside resistance
Efflux pumps	RND systems (AdeABC/AdeIJK, MexAB-OprM, MexCD-OprJ, MexEF-OprN, MexXY-OprM, AcrAB-TolC, OqxAB); MFS NorA	Dominant systems differ by pathogen: Mex systems in *P. aeruginosa*; Ade systems in *A. baumannii*; NorA in *S. aureus*	Regulated by AdeRS, MarA, SoxS, RamA, and local repressors
Fitness and compensation	Second-site substitutions, MarA/RamA/SoxS activation, clonal adaptations	*S. pneumoniae* shows fitness costs from QRDR substitutions; *P. aeruginosa* shows background-dependent costs	Compensatory changes preserve growth and maintain resistant lineages

### 3.4 Fitness costs and compensatory adaptations

**Figure 3:**
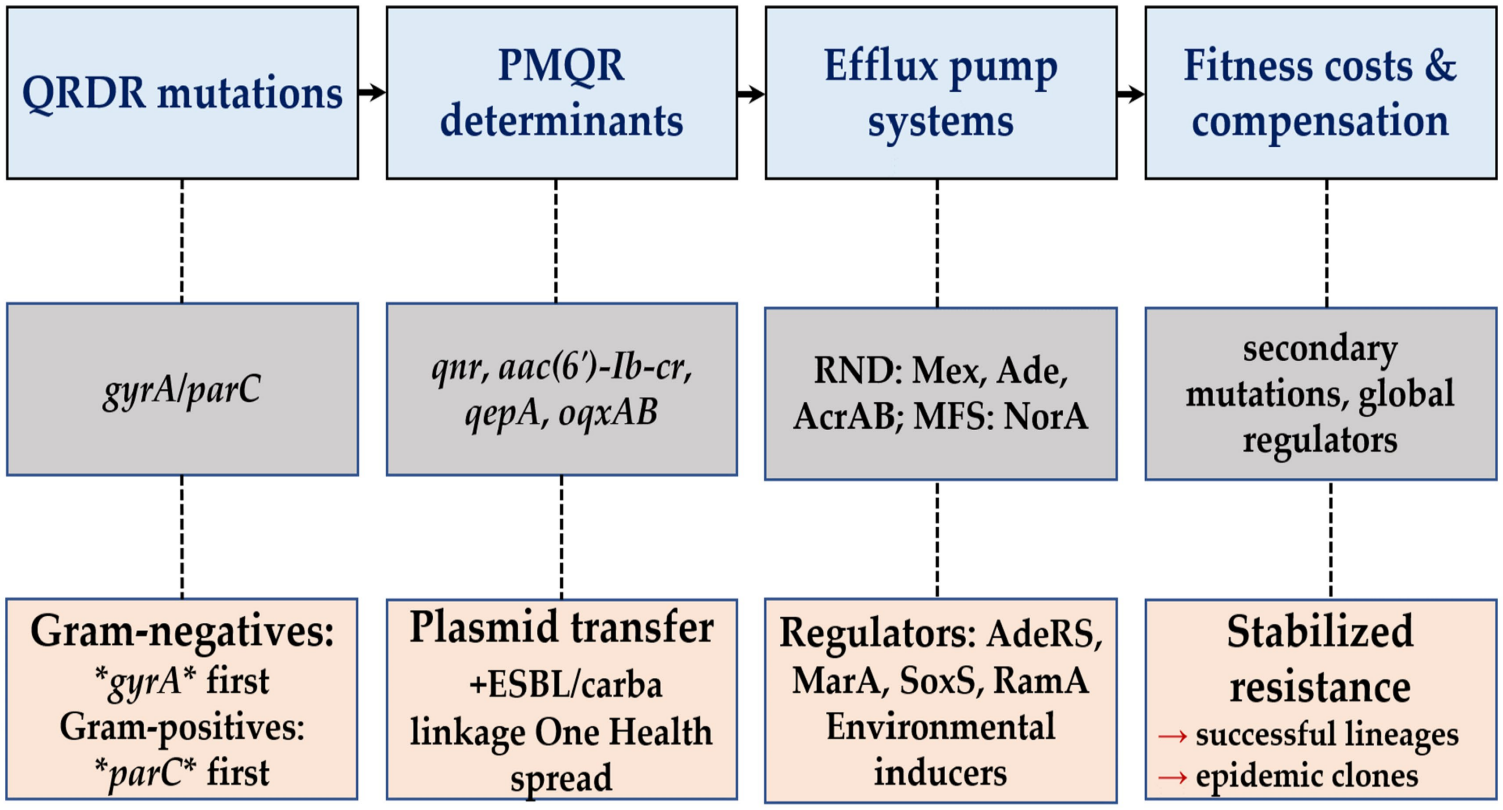
Molecular foundations of FQ-R in the ESKAPE pathogens. Resistance develops through substitutions in the QRDR of DNA gyrase and topoisomerase IV, acquisition of PMQR determinants such as *qnr*, *aac(6′)-Ib-cr*, *qepA*, and *oqxAB*, and overexpression of efflux pumps including the major facilitator superfamily protein NorA and RND systems such as AcrAB–TolC and Mex complexes. Species differ in target preference and reliance on efflux. Global regulators including *adeRS*, *marA*, *soxS*, and *ramA* shape expression of these mechanisms, while compensatory mutations reduce the fitness cost of resistance and support persistence of high-risk lineages.

Resistance often carries a biological cost that can impair growth, competitiveness, or transmission. Many bacteria develop compensatory changes that restore fitness while preserving resistance. In *Streptococcus pneumoniae* (*S. pneumoniae*), *in vitro* substitutions in *gyrA*, *parC*, and *parE* produced measurable fitness penalties, and the magnitude depended on the specific combination of mutations (Rozen et al., 2007). In Enterobacterales, laboratory evolution shows that second-site substitutions can offset these penalties; for example, a *parC* substitution can stabilize a low-fitness *gyrA* and *marR* background and further reduce susceptibility (Marcusson et al., 2009). Systems-level studies also show that induction of the global activators marA, ramA, and soxS enhances survival during antimicrobial stress by increasing efflux, but this shift reduces growth, biofilm formation, and virulence (Holden and Webber, 2020). Clinical and experimental work highlights strong lineage effects: resistance level and fitness cost vary by clonal background and regulatory genotype, suggesting that successful lineages have accumulated compensatory changes that minimize the biological burden of resistance (Agnello et al., 2016). Figure 3 summarizes how target mutations, PMQR determinants, efflux systems, global regulators, and fitness mechanisms interact across species.

These evolutionary patterns set the stage for several areas where the published literature remains inconsistent or difficult to reconcile. One recurring issue is the relative contribution of efflux and QRDR mutations. Many Enterobacterales collections follow a QRDR-first trajectory with early *gyrA* and *parC* substitutions, yet nonfermenters such as *P. aeruginosa* and *A. baumannii* often rely on early and intense efflux activation, with QRDR changes acting mainly as amplifiers in an efflux-primed background (Araya et al., 2023; Sakalauskienė et al., 2025). Another area of uncertainty involves PMQR. Studies of *qnr* families, *aac(6′)-Ib-cr*, and *oqxAB* describe heterogeneous fitness effects. Some experiments document measurable transmission or growth penalties, whereas others report little or transient cost because of compensatory evolution or the presence of low-cost plasmid backbones (San Millan and Maclean, 2017; Nohejl et al., 2022). Similar ambiguity surrounds the clinical significance of *oqxAB*. Although it originated on animal-associated plasmids and is common in food-animal and environmental isolates, reports differ on whether it contributes primarily low-level protection that becomes relevant only when combined with QRDR mutations or whether it plays a more direct role in clinical selection (Wang et al., 2017a; Amereh et al., 2023).

Additional variability arises from PD studies. MPC estimates and the width of the MSW vary widely between strains, laboratories, and assay conditions. This variability complicates direct translation of single-laboratory findings into universal dosing recommendations and underscores the need for standardized, context-specific application of these principles (Krajewska et al., 2023; Gianvecchio et al., 2019). Species-level differences also complicate interpretation. In Enterobacterales, porin loss is a major contributor to high-level resistance, whereas in *P. aeruginosa* intrinsic low permeability and strong RND efflux make porin remodeling less influential. These distinctions shape diagnostic priorities and stewardship strategies across pathogens (Sakalauskienė et al., 2025). These unresolved issues emphasize the need for harmonized methods and species-specific frameworks when interpreting FQ-R.

## 4 Evolutionary roadmaps in the ESKAPE pathogens

**Figure 4:**
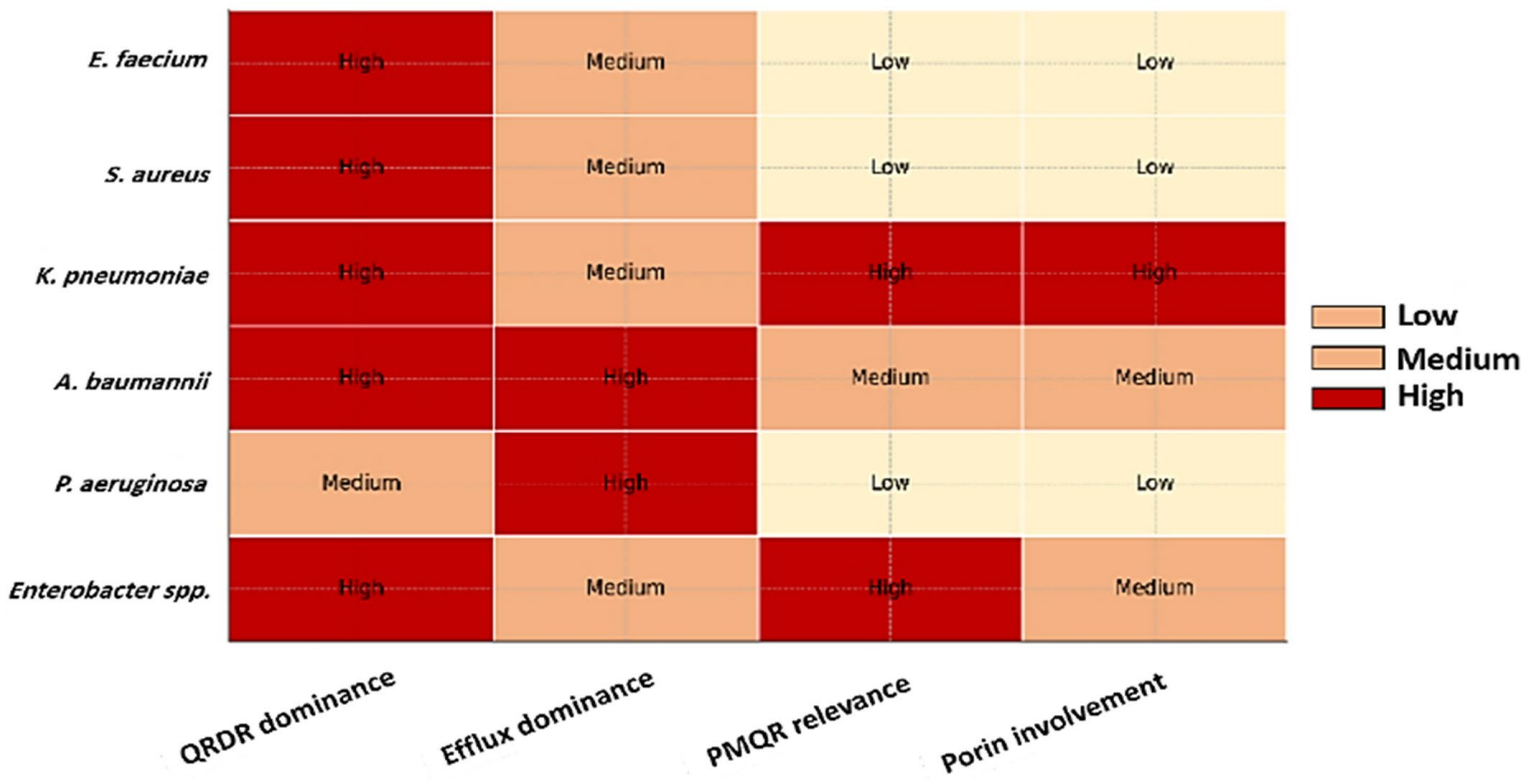
Species-wise comparison of FQ-R mechanisms across the ESKAPE pathogens. Heatmap showing the relative dominance of four core FQ-R mechanisms: QRDR mutations, efflux pump activity, PMQR, and porin remodeling. The comparison includes *E. faecium*, *S. aureus*, *K. pneumoniae*, *A. baumannii*, *P. aeruginosa*, and *Enterobacter* spp. Color shading reflects low, medium, or high contribution of each mechanism to clinically observed resistance phenotypes, based on consolidated genomic, molecular, and surveillance evidence presented in Section 4 and Table 3.

Building on the molecular modules in Section 3, this section outlines species-specific evolutionary paths across the ESKAPE group. Each roadmap integrates QRDR changes, PMQR elements, efflux regulation, porin remodeling, and compensatory adaptations, linking these mechanisms to clinically observed persistence and epidemiology. Figure 4 provides a species-level overview of the four principal FQ-R mechanisms, illustrating how the balance of QRDR mutations, efflux, PMQR, and porin changes varies markedly across the ESKAPE pathogens.

Because Section 3 provides the detailed mechanistic foundation for QRDR mutations, PMQR determinants, and efflux pathways, the roadmap descriptions below focus on species-level evolutionary trajectories and epidemiologic patterns rather than repeating molecular detail, thereby improving continuity and reducing redundancy across sections.

### 4.1 Enterococcus faecium

*Enterococcus faecium* shows poor intrinsic susceptibility to FQs, and clinical collections consistently report high nonsusceptibility. Genome-based surveys describe a broad accessory resistome and marked variability across healthcare-associated lineages, which limits genotype-only prediction (Coll et al., 2024; Werner et al., 2024). High-level resistance mainly reflects paired QRDR substitutions. In one cohort, 10 of 11 resistant isolates carried combined *gyrA* and *parC* changes, whereas a single low-level isolate lacking these substitutions likely depended on efflux or other topoisomerase changes (el Amin et al., 1999). Evidence from inhibitor studies indicates that efflux can raise MICs in some backgrounds, and its contribution varies by genotype (Mirzaii et al., 2023). Persistent circulation of hospital-adapted clones, especially clonal complex 17 enriched with mobile elements and multidrug traits, supports long-term survival and outbreak potential (Top et al., 2008). The combination of *gyrA/parC* substitutions, context-dependent efflux, and CC17-associated adaptation explains the sustained nonsusceptibility observed in hospital settings.

### 4.2 Staphylococcus aureus

In *Staphylococcus aureus*, resistance often follows a topoisomerase IV–first pattern. Substitutions typically arise in *parC/grlA*, including common GrlA S80F or S80Y and E84 variants, and later accumulate in *gyrA/gyrB*, producing stepwise increases in ciprofloxacin and norfloxacin MICs (Hooper and Jacoby, 2015; Truong-Bolduc et al., 2024; Huynh et al., 2023). Overexpression of Nor family pumps, particularly NorA, reduces intracellular FQ exposure and acts additively with QRDR changes (Hooper and Jacoby, 2015; Aeschlimann et al., 1999; Palazzotti et al., 2023; Truong-Bolduc et al., 2025). Expression of NorA, NorB, and NorC varies by clonal background and explains differences in MICs between lineages. In MRSA, healthcare-associated clones frequently harbor broader multidrug traits, whereas several community-associated lineages show efflux-driven decreases in susceptibility linked to altered NorA or NorB expression (Truong-Bolduc et al., 2024; Kwak et al., 2013; Sinha et al., 2024). This integration of QRDR changes with variable efflux regulation underpins the spectrum of resistance phenotypes seen across *S. aureus* populations.

### 4.3 Klebsiella pneumoniae

In *Klebsiella pneumoniae*, FQ nonsusceptibility commonly occurs alongside ESBL or carbapenemase plasmids, generating multidrug phenotypes with high outbreak potential. Surveys document convergence of QRDR mutations with PMQR determinants, including *qnr*, *aac(6′)-Ib-cr*, and *oqxAB*, which are often found together with *blaKPC*, *blaNDM*, or *blaOXA-48-like* (Li et al., 2024; Kherroubi et al., 2024; Han et al., 2020). Co-transfer of *qnr* with ESBL determinants and *aac(6′)-Ib-cr* has been confirmed through plasmid tracking, and PMQR determinants are frequent in FQ-nonsusceptible and ESBL-producing isolates across wards and specimen types (Kuo et al., 2024; Hoseinzadeh et al., 2024). Burn units also report high PMQR prevalence. Porin remodeling, frequently involving reduced expression or loss of OmpK35 or OmpK36, limits influx and, together with AcrAB–TolC and plasmid-mediated OqxAB, contributes to increased MICs and broad MDR or XDR phenotypes (Elías-López et al., 2024; Hussein et al., 2024). The spread of *blaNDM* or *blaOXA-48-like* plasmids that also harbor PMQR, or that arise in isolates with preexisting QRDR sets, contributes to substantial FQ-R in carbapenem-resistant strains and emphasizes the importance of integrated stewardship and infection-prevention measures (Lazar et al., 2024; Braun et al., 2024).

### 4.4 Acinetobacter baumannii

Clinical *A. baumannii* follows a trajectory in which RND efflux and QRDR mutations combine to elevate MICs and maintain MDR under ICU conditions (Roy et al., 2021; Mohammed et al., 2021; Kyriakidis et al., 2021). Functional analyses show that AdeABC, AdeFGH, and AdeIJK have distinct and complementary roles, with inactivation of individual pumps yielding substrate-specific effects (Xie et al., 2025). Transcriptomic work supports nonoverlapping responses of AdeABC and AdeIJK, which aligns with their different contributions to intrinsic adaptation (Wei et al., 2024). AdeIJK is widespread and forms the baseline for intrinsic nonsusceptibility, whereas AdeABC functions as an acquired amplifier whose overexpression, generally linked to *adeRS* dysregulation, increases FQ and broad-spectrum MICs (Darby et al., 2023; Saral Sariyer et al., 2025). QRDR mutations in *gyrA*, *gyrB*, and *parC* are frequent in high-level resistance and often coexist with efflux alterations (Park et al., 2011). These features mirror ICU ecology, where prolonged environmental survival, resistance islands, and international clone 2 support long-term persistence and outbreaks (Fonseca et al., 2023; Hamed et al., 2023). In summary, AdeIJK establishes the intrinsic baseline, AdeABC augments clinical MDR under *adeRS*, and accumulated QRDR changes complete an FQ-resistant, outbreak-prone profile (Xie et al., 2025; Wei et al., 2024; Park et al., 2011; Boonsilp et al., 2024).

### 4.5 Pseudomonas aeruginosa

*Pseudomonas aeruginosa* combines high intrinsic tolerance with rapid adaptive capacity. Resistance usually begins with QRDR substitutions in *gyrA* and *parC*, classically GyrA T83I and ParC S87L, with additional substitutions further increasing MICs (Arefin et al., 2025; Zhao et al., 2020). RND efflux evolves in parallel. MexAB-OprM, MexCD-OprJ, MexEF-OprN, and MexXY-OprM mediate extrusion, and MexAB-OprM typically dominates clinically. Regulatory mutations in *mexR*, *nalC*, or *nfxB* often drive overexpression (Llanes et al., 2011; Boushra et al., 2024). Efflux augments QRDR effects and moves MICs beyond clinical breakpoints, and efflux inhibition slows the multistep path to high-level resistance (Yu et al., 2025).

Mobilizable determinants such as *crpP* on integrative and conjugative elements add low-level resistance that can interact with chromosomal mechanisms (López et al., 2022). In ventilator-associated pneumonia (VAP), UTI, and cystic fibrosis, isolates commonly harbor both QRDR changes and efflux upregulation, producing stable MDR in nosocomial lineages (Boushra et al., 2024; Cabot et al., 2016). Table 4 summarizes the principal mechanisms and clinical implications.

**Table 4:** Mechanisms of FQ-R in *P. aeruginosa.*

Mechanism	Gene/mutation	Clinical impact	References
QRDR mutations	*gyrA* Thr83 to Ile; *parC* Ser87 to Leu	Associated with high-level resistance and progressive MIC elevation	Arefin et al. (2025), Zhao et al. (2020), and López et al. (2022)
Efflux pump overexpression	MexAB-OprM, MexCD-OprJ, MexEF-OprN, MexXY-OprM; regulators mexR, nalC, nfxB	Acts with QRDR mutations to raise MICs above clinical breakpoints	Llanes et al. (2011) and Boushra et al. (2024)
Experimental inhibition of efflux	Targeting MexAB-OprM and related systems	Slows or prevents stepwise evolution to high-level resistance	Yu et al. (2025)
Mobile resistance elements	*crpP* on integrative and conjugative elements and plasmids	Adds low-level resistance and interacts with chromosomal routes	López et al. (2022)
Clinical persistence	Combined QRDR mutations, efflux activity, and mobile determinants	Maintains MDR in VAP, UTIs, and cystic fibrosis isolates	Boushra et al. (2024) and Cabot et al. (2016)

### 4.6 *Enterobacter* spp

In *Enterobacter* spp., FQ-R usually develops through chromosomal QRDR substitutions combined with plasmid-mediated mechanisms under hospital selection pressure. Clinical and molecular investigations consistently report *gyrA* substitutions, often at codon 83, and *parC* substitutions such as S80 to I, many of which occur together with PMQR determinants (Kotb et al., 2019; Albornoz et al., 2017). A whole-genome survey of Enterobacteriaceae, including *Enterobacter* spp., showed that high-level resistance, with MICs ranging from 4 to 512 μg/mL, was frequently driven by multiple QRDR mutations. Plasmid-mediated *oqxAB*, *aac(6′)-Ib-cr*, and *qnr* variants were also identified, demonstrating parallel chromosomal and plasmid routes to resistance (Osei Sekyere and Amoako, 2017).

PMQR is well documented in hospital collections. *qnrB*, *qnrS*, and *aac(6′)-Ib-cr* often co-localize with *β*-lactam resistance determinants on conjugative plasmids (Cano et al., 2009). Hospital outbreaks of FQ-R *Enterobacter* frequently involve strains that already exhibit β-lactam resistance, and the combination of QRDR substitutions with PMQR elements supports persistence during therapy and facilitates intra-ward transmission (Kotb et al., 2019; Piekarska et al., 2021).

These findings outline a dual-pathway model in which incremental chromosomal mutations provide the vertical framework of resistance, while PMQR plasmids supply horizontal reinforcement. This combination stabilizes high-level resistance and supports clonal expansion in hospital environments. Across the ESKAPE pathogens, these species-specific pathways converge on a shared pattern in which QRDR substitutions, shaped by PMQR and strengthened by efflux and porin remodeling, are conditioned by clonal background and local plasmid ecology.

## 5 PK/PD as evolutionary drivers

### 5.1 PK/PD principles shaping evolution

Antibiotic exposure determines which mutants persist, meaning PK/PD directly governs evolutionary selection. The MSW spans concentrations between the wild-type MIC and the MPC (Drlica and Zhao, 2007). Within this interval, first-step mutants gain a selective advantage, whereas exposures at or above the MPC suppress amplification (Drlica and Zhao, 2007). Recent work distinguishes predominant from inferior mutants and proposes metrics such as MPC-D and MSW-D to better estimate *in vivo* risk (Krajewska et al., 2023). In practice, early high exposure that maximizes Cmax and AUC relative to MIC or MPC limits enrichment when bacterial burden is greatest (Drlica and Zhao, 2007; Krajewska et al., 2023).

For FQs and DPAs, the 24-h AUC/MIC remains the most reliable index for efficacy and suppression of resistance (Forrest et al., 1993; Ambrose et al., 2001). In severe infections, higher AUC/MIC correlates with more rapid clearance, supporting ciprofloxacin targets of total-drug AUC/MIC at or above 125, which corresponds to fAUC/MIC around 90 for Gram-negative pathogens (Forrest et al., 1993; Abdulla et al., 2020). Standard regimens often fail to reach these thresholds for contemporary MICs of about 0.25 to 0.5 mg/L, resulting in prolonged time within the MSW (Haeseker et al., 2013; de Vroom et al., 2020). In hospitalized adults, this shortfall is common; simulations suggest that approximately 1,200 mg per day intravenously may be needed in patients with normal renal function (Haeseker et al., 2013). Prospective ward studies confirm frequent nonattainment even after renal adjustment, indicating the need for individualized dosing (De Vroom et al., 2020).

For *S. pneumoniae*, targets are lower. Hollow-fiber and translational studies with levofloxacin indicate fAUC/MIC of roughly 30 to 50 (Ambrose et al., 2001; Lacy et al., 1999). High-exposure regimens, such as 750 mg every 24 h for community-acquired pneumonia, typically achieve these levels. Subthreshold exposure extends time within the MSW and delays sterilization (Ambrose et al., 2001; Lacy et al., 1999). Although Cmax/MIC may predict certain endpoints, AUC/MIC remains the most consistent measure when suppression of resistance is the goal (Ambrose et al., 2001).

These data support several practical steps. First, maximize target attainment by integrating MICs, renal function, body size, and, when available, Bayesian AUC estimates (De Vroom et al., 2020; Forrest et al., 1993). Second, set policy according to updated breakpoints, since outcomes decline when MICs cluster near former susceptible thresholds, indicating covert underdosing (Van et al., 2019). Third, use front-loaded exposure when feasible to achieve early bactericidal activity before narrowing therapy or stopping once microbiologic data permit (Drlica and Zhao, 2007). Fourth, limit duration to reduce microbiome selection and pair therapy with exposures that minimize time within the MSW (Jakobsson et al., 2010). Two caveats apply: variability in MIC and MPC assays can distort target estimates, and differences between plasma and site concentrations, such as epithelial lining fluid, can modify effective exposure. These limitations reinforce the value of individualized, PK-guided dosing (Jakobsson et al., 2010).

### 5.2 A unified PK/PD evolution and clonal expansion model

FQ-R follows a unified pathway linking pharmacology, molecular evolution, and clonal expansion. Standard ciprofloxacin regimens frequently fail to meet AUC/MIC targets (Haeseker et al., 2013; Sassen et al., 2020), which leaves patients exposed to concentrations that fall within the MSW. In this range, first-step mutants are selectively favored, and low-level mechanisms such as *qnr*, *aac(6′)-Ib-cr*, *oqxAB*, modest efflux activation, and single QRDR substitutions each confer incremental survival. These small advantages broaden the functional MSW and promote accumulation of additional chromosomal changes or mobile elements (Amereh et al., 2023; Chen et al., 2024).

Experimental evidence shows that efflux upregulation can markedly amplify the effect of QRDR mutations. For example, MexAB-OprM combined with *gyrA* substitutions in *P. aeruginosa* produces MIC increases far greater than either mechanism alone (Nakajima et al., 2002; Langendonk et al., 2021). Over time, plasmid- or stress-induced compensatory adaptations stabilize the fitness of these initially costly genotypes. This stabilization enables successful clones, including *E. coli* ST131, *A. baumannii* IC2/ST2, and *P. aeruginosa* ST235, to expand across healthcare networks (Chen et al., 2024; Melnyk et al., 2015).

These relationships connect within-host PK/PD to population-level epidemiology. Suboptimal exposure drives early mutational steps, PMQR and efflux reinforce survival within the MSW, and compensatory evolution secures competitive fitness. These processes generate high-risk lineages that spread regionally and globally.

## 6 Clonal expansion and global dissemination

FQ-R becomes epidemiologically relevant when molecular changes described in earlier sections merge with high-risk clonal backgrounds. These clones carry stable combinations of QRDR substitutions, PMQR determinants, and efflux traits, and they spread efficiently across healthcare networks and international borders. Section 6 synthesizes these population-level patterns by focusing on clones and plasmids that sustain long-term transmission.

### 6.1 High-risk clones across the ESKAPE pathogens

Across the ESKAPE group, a limited number of lineages account for a large proportion of resistant infections. In *K. pneumoniae*, clonal group CG258, especially ST258, represents the best-known blaKPC-associated lineage. Genomic work dates its expansion to the mid-1990s and links its success to pKpQIL-like IncFIIK plasmids (Bowers et al., 2015; Doumith et al., 2017). Other successful lineages, including ST147, ST307, and ST11, have repeatedly caused international and regional outbreaks and frequently carry ESBLs and carbapenemases (Zhang et al., 2025a).

In *A. baumannii*, international clones IC1 and IC2 (largely ST1 and ST2) dominate global CRAB epidemiology and remain the leading cause of hospital outbreaks in many regions (Müller et al., 2023; Nodari et al., 2020). In *P. aeruginosa*, ST235, ST111, and ST175 are widely distributed high-risk clones. ST235 in particular is associated with multiple carbapenemases and exoU-linked virulence, while ST111 has been identified across environmental and clinical reservoirs (del Barrio-Tofiño et al., 2020; Correa et al., 2015).

In *E. faecium*, CC17 continues to define the hospital-adapted lineage enriched in resistance determinants, colonization traits, and genomic islands associated with VRE spread (Top et al., 2008; Top et al., 2008). Within the *Enterobacter cloacae* complex, ST171 and ST78 recur across continents in the emergence of carbapenem-resistant Enterobacter and contribute to sustained healthcare transmission (Gomez-Simmonds et al., 2018; Ye et al., 2025; Zeng et al., 2024).

### 6.2 Mobile elements and plasmid dissemination

Clonal success is reinforced by mobile elements that transfer key resistance determinants within and between species. In *K. pneumoniae* CG258, pKpQIL-like IncFIIK plasmids were central to the early expansion of blaKPC and continue to circulate across the species complex and into other Enterobacterales (Doumith et al., 2017; Chen et al., 2014; Buckner et al., 2018). For OXA-48-type carbapenemases, global dissemination has been driven by a conserved IncL plasmid backbone of approximately 60 to 63 kb with high conjugation efficiency and low fitness cost (Lee et al., 2016; David et al., 2020; Hamprecht et al., 2019).

blaNDM variants, particularly blaNDM-5 and blaNDM-7, are frequently carried by IncX3 plasmids with broad host ranges and strong stability. Clinical and experimental work has documented transmission across species and even between different bacterial phyla (Tian et al., 2020; Tao et al., 2023; Yang et al., 2024). Over time, the genetic context surrounding blaNDM has diversified, as seen in the evolution from Tn125 to IS26-derived structures and then to Tn3000. These transitions have shaped regional plasmid dominance (Acman et al., 2022). In *A. baumannii*, resistance islands such as AbaR and class 1 integrons reinforce MDR phenotypes within IC1 and IC2, facilitating rapid adaptation to ICU antimicrobial pressure (Müller et al., 2023). Together, high-risk clones and adaptable plasmid backbones create a modular architecture that favors persistence and repeated re-emergence.

### 6.3 Cross-border and healthcare-associated spread

Once established, these clones disseminate through referral networks, patient transfers, shared healthcare pathways, and medical tourism. ST258 within CG258 spread across continents within a few years, supported by hospital transmission and stable pKpQIL-like plasmids (Bowers et al., 2015; Doumith et al., 2017). The *P. aeruginosa* complex containing ST235, ST111, and ST175 has expanded across Europe, Asia, and the Americas in VAP, bloodstream infections, and UTIs (del Barrio-Tofiño et al., 2020; Correa et al., 2015).

In *E. faecium*, CC17 has become the dominant global VRE lineage by combining colonization capacity with survival advantages on hospital surfaces (Top et al., 2008). Plasmids themselves also move across borders and species. The IncL pOXA-48a backbone has disseminated widely in *K. pneumoniae*, *E. coli*, and *Enterobacter* spp. (Lee et al., 2016; Pulss et al., 2018). IncX3 plasmids continue to spread blaNDM-5 and blaNDM-7 internationally (Tian et al., 2020; Harada et al., 2024). Continued surveillance documents regional expansion of ECC ST171 and ST78 across several continents, often linked to patient movement (Gomez-Simmonds et al., 2018; Zeng et al., 2024).

Across the ESKAPE pathogens, global dissemination reflects the convergence of a small set of highly adapted clonal backgrounds CG258 in *K. pneumoniae*, IC1 and IC2 in *A. baumannii*, ST235 and related lineages in *P. aeruginosa*, CC17 in *E. faecium*, and ST171 and ST78 in ECC with efficient plasmids such as pKpQIL-like IncFIIK carrying blaKPC, the conserved IncL pOXA-48a carrying blaOXA-48, and IncX3 plasmids transmitting blaNDM (Bowers et al., 2015; del Barrio-Tofiño et al., 2020). This joint movement of clones and plasmids underscores the need for molecular surveillance that captures both components and links them to infection-prevention practice.

## 7 One health dimensions

FQ-R develops and circulates across interconnected human, animal, and environmental reservoirs. The molecular and clonal trajectories outlined in earlier sections are reinforced by selection pressures and gene flow outside healthcare settings. Current One Health frameworks increasingly view these nonclinical reservoirs as essential drivers of PMQR maintenance and transmission.

### 7.1 Role of agriculture, veterinary medicine, and environmental reservoirs

Selection pressures in food-animal production play a central role in sustaining FQ-R beyond clinical settings. Veterinary quinolone use, including agents such as enrofloxacin, creates favorable conditions for the acquisition and long-term maintenance of PMQR determinants, including *qnr* families, *aac(6′)-Ib-cr*, *qepA*, and *oqxAB*. These determinants frequently co-localize with extended-spectrum *β*-lactamase or AmpC genes on conjugative plasmids, enabling coordinated selection under multiple antimicrobial exposures (Gomi and Adachi, 2025; Chen et al., 2024; Chen et al., 2012; Zhao et al., 2010).

Direct evidence from poultry–human interface studies further illustrates how these selection pressures translate into human exposure. In a comparative investigation of *Campylobacter jejuni* isolated from broilers, laying hens, and farm workers, high levels of FQ-R were detected across all sectors, with ciprofloxacin resistance exceeding 68% and frequent detection of gyrA exceeding 75% (Shami et al., 2024). The simultaneous recovery of resistant strains from poultry and individuals working on the same farms provides a clear example of food-chain–associated transmission and highlights how veterinary FQ use can select resistant bacteria that subsequently reach humans outside hospital settings.

Similar patterns of selection and transmission are evident in dairy production systems. Investigations of white soft cheese and yoghurt have revealed the presence of MDR *S. aureus*, *E. coli*, and *P. aeruginosa*, including ESKAPE-associated species carrying both resistance and virulence determinants. These observations suggest that dairy products can function as downstream reservoirs of AMR bacteria, connecting AMU on farms with food-processing environments and subsequent human exposure within a unified One Health framework (Nadi et al., 2024).

Hatchery environments represent an additional amplification point within poultry production systems. Environmental investigations have shown that *P. aeruginosa* can persist on hatchery surfaces, in air samples, and within dead embryos, indicating that insufficiently controlled hatchery hygiene allows zoonotic pathogens to be maintained and further disseminated along the production chain beyond the farm level (Ibrahim et al., 2024).

Experimental work in veterinary settings has demonstrated that green-synthesized selenium nanoparticles can disrupt *S. aureus* biofilms and reduce bacterial burden in mastitis models, highlighting the potential of nanomaterial-based strategies as adjunct measures to reduce transmission pressure at the animal and environment interface (Ibrahim et al., 2024). These findings are consistent with broader veterinary and One Health syntheses identifying nanoparticle-based approaches as promising non-antibiotic tools for limiting biofilm persistence and enhancing infection control in animal production systems (Hannan et al., 2024).

In parallel with nanomaterial-based approaches, natural antimicrobial compounds have also been explored as alternative tools in veterinary settings. Extracts from the filamentous green alga *Spirogyra neglecta* have demonstrated inhibitory activity against mastitis-associated pathogens, including *S. aureus*, *E. coli*, and *P. aeruginosa*, supporting the potential role of plant- and algae-derived agents in reducing bacterial burden and downstream transmission pressure in animal production systems (Insumran et al., 2024).

A defining feature of this process is the movement of resistance determinants across human, animal, and environmental interfaces. Comparative genomic studies consistently show that similar PMQR-bearing plasmid backbones circulate among *E. coli* from livestock, food products, environmental sources, and human populations, supporting bidirectional transmission rather than isolated sector-specific evolution (Chen et al., 2012; Zhao et al., 2010).

Aquatic environments function as critical ecological connectors within this network. Wastewater and surface waters receiving agricultural and municipal effluents facilitate mixing of bacterial populations and mobile genetic elements, allowing resistance determinants selected in one sector to persist and re-enter others. The WHO Tricycle protocol, which uses ESBL-producing *E. coli* as a standardized One Health indicator organism, demonstrates the feasibility of tracking these transmission pathways across sectors and provides a framework that can be readily extended to fluoroquinolone resistance markers (Milenkov et al., 2024; Bivins et al., 2025).

Large-scale environmental surveillance further reinforces the role of non-clinical reservoirs in sustaining and disseminating AMR. A nationwide investigation across multiple Lebanese districts identified widespread multidrug-resistant Gram-negative ESKAPE pathogens, including *E. coli*, *K. pneumoniae*, *P. aeruginosa*, and *A. baumannii*, in water, sewage, soil, and animal samples. Whole-genome sequencing revealed clinically relevant high-risk clones, plasmid-mediated resistance genes, efflux systems, and conserved FQ-R mechanisms, demonstrating that environmental compartments function as active reservoirs and mixing points for resistance determinants with direct relevance to human health (Samadi et al., 2025).

These conceptual links underscore why FQ-R cannot be contained through hospital stewardship alone. Instead, resistance trajectories reflect interconnected selection pressures operating across agriculture, the environment, and clinical care, a pattern reinforced by coordinated European surveillance showing that reductions in veterinary quinolone use are followed by measurable declines in resistance among food-animal isolates (European Centre for Disease Prevention and Control, European Food Safety Authority, European Medicines Agency, 2017). Section 7.2 and Table 5 summarize how these selective pressures manifest quantitatively across environmental compartments.

**Table 5:** One health AMU and AMR correlations.

Sector	Representative AMU/metric	Verified FQ-R/PMQR prevalence (range)	Key insight	Ref.
Poultry (food animals)	Quinolone sales aggregated (ESVAC 2022): mean quinolone sales ~2.2 mg/PCU across reporting EU countries (quinolones include FQs).	PMQR detection in poultry/swine studies varies by region; representative reported ranges in multi-country studies: ~20–50% PMQR positivity; specific porcine collections reported oqxAB ~40–50%.	Poultry and swine are important reservoirs for oqxAB and other PMQR genes; elevated veterinary use correlates with higher sectoral FQ-R in multiple surveillance analyses.	Chen et al. (2012), European Medicines Agency (2023), and Wang et al., (2017b)
Swine (food animals)	Included in species-aggregated ESVAC mg/PCU national sales	PMQR/oqxAB prevalence in porcine isolates reported ~20–50% in regional/multicenter studies; some Chinese datasets report ~40–50% oqxAB in swine isolates.	Swine frequently carry oqxAB-positive plasmids; co-location with ESBL plasmids promotes co-selection under AMU.	Chen et al. (2012), European Medicines Agency (2023), and Wang et al. (2017b)
Cattle (food animals)	ESVAC reports species-aggregated sales; quinolone use often lower in cattle than in poultry/swine (country-dependent).	Lower PMQR/FQ-R in many surveys compared with poultry/swine; representative range across studies ~5–25%.	Cattle show intermediate resistance burdens consistent with lower relative quinolone use.	European Medicines Agency (2023) and European Food Safety Authority, European Centre for Disease Prevention and Control, European Medicines Agency (2021)
Humans (community isolates)	Human community consumption varies by country; GLASS/JIACRA report DDD metrics and community prescribing data.	ECDC/EARS-Net 2024: population-weighted mean FQ resistance across reporting EU/EEA countries ~15% for monitored species groups; country heterogeneity with some settings >40–50% for specific pathogens or specimen types.	Community AMU contributes to community FQ-R; travel and cross-sector transmission amplify heterogeneity.	European Centre for Disease Prevention and Control (2025), World Health Organization WHO (2024), and European Food Safety Authority, European Centre for Disease Prevention and Control, European Medicines Agency (2021)
Humans (hospital isolates)	Hospital (in-patient) FQ use is higher and targeted; stewardship programs shape local consumption.	FQ-R in nosocomial pathogens (e.g., *K. pneumoniae*) often exceeds community rates; pockets >50% reported in several countries/settings.	Hospital selection pressure and clonal expansion drive elevated FQ-R in nosocomial pathogens.	European Centre for Disease Prevention and Control (2025) and World Health Organization (2024)
Wastewater influent (urban / hospital)	Used as sentinel for upstream contamination; reported as gene copies/mL or % PMQR-positive isolates in studies.	PMQR detection in influent/wastewater studies shows wide variation by region and method; compiled ranges across studies ~20–66% PMQR-positive (site-dependent).	Wastewater is an enrichment node for PMQR and ESBL genes and reflects upstream AMU and carriage.	Amin et al. (2021)
Surface / treated water (downstream rivers, lakes, tap water)	Lower ARG/PMQR abundance than raw influent; usually reported as % PMQR-positive isolates or ARG gene copies per mL.	PMQR genes frequently detected in surface and treated waters, but at reduced prevalence vs. influent; many studies report single- to low-double-digit % PMQR positive Enterobacterales, with higher values in heavily impacted sites.	Treatment substantially reduces but does not eliminate PMQR/ARGs; residual contamination allows continued downstream environmental dissemination.	Yan et al. (2017) and Liguori et al. (2022)

Representative sectoral AMU metrics, verified FQ-R / PMQR prevalence ranges, and key insights.

### 7.2 Quantitative one health trends in AMU and AMR dynamics

Cross-sector surveillance data provide quantitative evidence that AMU directly shapes FQ-R across human, animal, and environmental reservoirs. Joint JIACRA and EFSA/ECDC analyses consistently associate higher veterinary FQ consumption with increased resistance in *E. coli* isolated from both food animals and humans. ESVAC data report an average quinolone use of approximately 2.2 mg per population correction unit across Europe, with marked regional variation that parallels national resistance patterns (European Medicines Agency, European Food Safety Authority, and European Centre for Disease Prevention and Control, 2024; European Centre for Disease Prevention and Control, European Food Safety Authority, European Medicines Agency, 2021; European Medicines Agency, 2023).

Molecular surveillance further demonstrates that PMQR determinants are unevenly distributed across reservoirs. *Qnr*, *aac(6′)-Ib-cr*, *qepA*, and *oqxAB* are most frequently detected in *E. coli* and *K. pneumoniae* from animal and environmental sources, where reported detection rates commonly range from 10–40% for *E. coli* and 5–25% for *K. pneumoniae*, depending on geography and sampling design (Chen et al., 2012; Park et al., 2006; Li et al., 2019). These values often exceed those observed in invasive human isolates, consistent with agricultural enrichment and food-chain transmission. Systematic One Health reviews confirm that food and environmental reservoirs consistently exhibit higher baseline carriage of resistance determinants than clinical samples, supporting their role as upstream sources (Oyenuga et al., 2024; Khasapane et al., 2024).

Recent integrative genomic syntheses further highlight that quantitative AMR trends across these reservoirs can only be interpreted accurately through coordinated One Health surveillance frameworks that combine AMU data with high-resolution genomic and metagenomic analyses, enabling detection of both clonal expansion and horizontal gene transfer across human, animal, and environmental interfaces (Liu and Pandey, 2024).

Environmental monitoring complements these findings. Influent wastewater and runoff from livestock operations frequently contain PMQR determinants at prevalences ranging from approximately 20% to more than 60%, whereas treated effluents and downstream surface waters show lower but persistent levels, reflecting incomplete removal during wastewater treatment (Zou et al., 2021; Amin et al., 2021; Ranjbar and Farahani, 2017). These patterns are consistent with EFSA assessments identifying aquatic systems as long-term reservoirs and mixing points for antimicrobial resistance genes entering human and food-chain exposure pathways (Koutsoumanis et al., 2021).

Longitudinal MIC data mirror these quantitative trends. Many Enterobacterales collections show a gradual right-shift in ciprofloxacin MIC distributions from the 1990s through the 2010s, coinciding with expansion of PMQR determinants and globally disseminated FQ-R clones such as *E. coli* ST131. In contrast, countries implementing coordinated reductions in veterinary FQ use demonstrate stabilization or modest declines in resistance over time [Chen et al., 2012; European Medicines Agency (EMA), 2024]. These observations support evolutionary models describing resistance emergence as a population-level response to sustained antimicrobial pressure rather than isolated clinical events (Baquero et al., 2021).

These findings support the use of integrated GLASS-AMR, GLASS-AMC and One Health dashboards that pair AMU metrics with resistance trajectories and allow early identification of settings where targeted interventions are most effective. Table 5 summarizes representative AMU metrics and verified FQ-R/PMQR prevalence across major One Health reservoirs.

### 7.3 Transmission of PMQR and FQ-resistant clones beyond hospitals

FQ-R clones and PMQR determinants circulate widely outside hospitals, reflecting the continuity between human, animal, and environmental reservoirs described earlier. Companion-animal studies show that dogs can carry and shed FQ-R *E. coli* in the same geographic settings where similar resistant strains and PMQR profiles are detected in humans and cattle, indicating shared exposures and local cross-species transmission pathways (Sealey et al., 2023).

Across sectors, identical PMQR constellations (*qnr*, *aac(6′)-Ib-cr*, *qepA*, *oqxAB*) appear in clinical, agricultural, and environmental isolates, typically embedded in mobile genetic platforms that facilitate efficient horizontal spread. These determinants confer low-level protection that supports survival at intermediate drug concentrations and allows QRDR mutations to accumulate, enabling resistant clones to become established in community reservoirs (Gomi and Adachi, 2025; Chen et al., 2012; Zhao et al., 2010).

Integrated surveillance efforts that combine human AMR data (GLASS-AMR), antimicrobial-use data (GLASS-AMC), and interagency analyses such as JIACRA consistently show that higher quinolone use correlates with higher FQ-R in both human and animal *E. coli*. These programs also document bidirectional transmission of PMQR elements and FQ-R lineages across hospitals, farms, food chains, and community settings (Ajulo and Awosile, 2024; European Centre for Disease Prevention and Control, European Food Safety Authority, European Medicines Agency, 2017).

### 7.4 Integrated surveillance needs

Effective control of FQ-R requires surveillance systems that align AMR and AMC indicators across human, veterinary, and environmental arenas. National programs should report GLASS-AMR and GLASS-AMC together so that resistance trends can be directly evaluated against drug-use patterns and stewardship policies [World Health Organization (WHO), 2015]. Building on the WHO Tricycle framework, an expanded FQ module should include QRDR genotyping (World Health Organization, 2021), targeted PMQR screening (*qnr*, *aac(6′)-Ib-cr*, *qepA*, *oqxAB*), and plasmid typing (IncX/IncX1, IncF, IncL) to resolve gene, vector, and host relationships and identify shared plasmid backbones across sectors. JIACRA IV and related interagency reports show how harmonized AMC and AMR data support targeted interventions and enable assessment of stewardship programs (European Centre for Disease Prevention and Control, European Food Safety Authority, European Medicines Agency, 2017; European Centre for Disease Prevention and Control, European Food Safety Authority, European Medicines Agency, 2024).

Environmental components are equally important. Standardized wastewater and surface-water sampling at hospital outlets, food-production sites, and downstream recreational areas, combined with WGS, can map ESBL and PMQR reservoirs and quantify their persistence (Liguori et al., 2022; Punch et al., 2025; Al-Mustapha et al., 2025). Actionable surveillance should detect AMC and AMR discordance (for example, increasing FQ resistance despite stable or declining use) and provide cross-sector dashboards to evaluate intervention impact.

High-flux interfaces international travel, imported food products, and companion-animal contact require dedicated monitoring because they allow PMQR determinants and FQ-R lineages to move rapidly between settings. Multiple One Health studies show circulation of *E. coli* across these interfaces, reinforcing their relevance for early detection and control (Sealey et al., 2023; Umeda et al., 2020; Cui et al., 2022).

**Figure 5:**
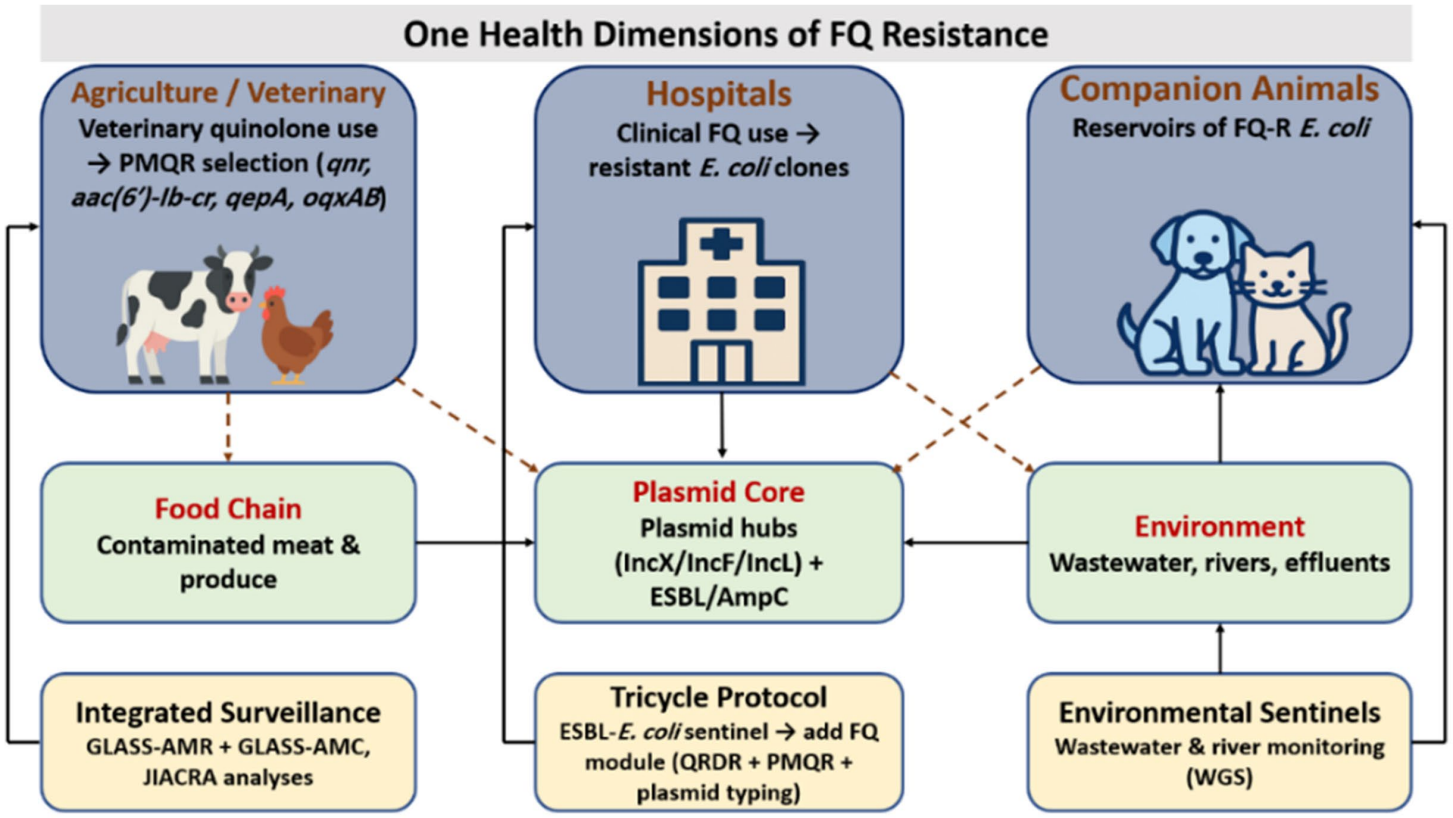
One health dimensions of FQ-R. Agriculture, veterinary care, hospitals, and companion animals act as interconnected sources of selection and reservoirs of FQ-R *E. coli*. Veterinary quinolone use promotes the emergence of PMQR determinants (*qnr*, *aac(6′)-Ib-cr*, *qepA*, *oqxAB*), while clinical use in hospitals selects for resistant clones. These determinants move through food chains and environmental waters, supported by plasmid backbones (IncX, IncF, IncL) that often carry ESBL or AmpC. Integrated surveillance systems, including GLASS-AMR/AMU, the WHO tricycle protocol, and environmental sentinels, provide cross-sector monitoring and support harmonized one health strategies to limit FQ-R.

Overall, synchronized reductions in FQ pressure, combined with plasmid-aware genomic surveillance focused on key mobile elements (*qnr*, *aac(6′)-Ib-cr*, *qepA*, *oqxAB*), remain central to limiting spread (Vázquez et al., 2022). As shown in Figure 5, plasmid hubs link agricultural, environmental, and clinical reservoirs and underscore the need for integrated systems such as GLASS-AMR/AMC, the WHO Tricycle framework, and coordinated environmental sentinels to detect and disrupt transmission across sectors.

Table 6 summarizes the major research gaps and priority questions across molecular, clinical, and One Health domains. These gaps include uncertainties related to interactions between efflux and QRDR pathways, the fitness effects of PMQR determinants, the relationship between porin remodeling and efflux, the need for standardized MSW and MPC methodologies, the dynamics of clonal evolution, the routes of plasmid transmission, and the readiness of emerging approaches such as efflux inhibitors, plasmid-transfer blockers, and AI-based prediction tools. These issues provide a forward-looking roadmap to guide future investigations and to strengthen surveillance, stewardship, and therapeutic development efforts.

**Table 6:** Research gaps and priority questions in FQ-R across ESKAPE pathogens.

Research gap	Description of unresolved issue	Priority questions
Efflux versus QRDR dominance by species	Relative contribution of efflux versus target mutations varies widely; conflicting data on which mechanism dominates early resistance.	What factors determine efflux- or QRDR-first evolution? Can predictive models be developed for species-specific trajectories?
PMQR fitness cost variability	PMQR determinants show heterogeneous fitness effects across lineages and plasmid backbones.	When do PMQR genes impose real fitness burdens? Which plasmid features minimize these costs?
Uncertain role of oqxAB in clinical settings	OqxAB common in agriculture and environment, but its clinical significance is unclear without QRDR/efflux changes.	How significant is oqxAB in clinical failures? Should it be included in routine surveillance?
Non-standardized MSW/MPC methodologies	Wide variability in MSW and MPC data between labs and strains.	Can internationally standardized MPC/MSW protocols be developed? How to validate MSW for clinical dosing?
Porin-efflux interplay not fully defined	Porin loss significant in Enterobacterales but less relevant in *P. aeruginosa* where RND efflux dominates.	What frameworks can predict MIC shifts incorporating permeability + efflux + QRDR effects?
Plasmid flow across One Health sectors	PMQR/ESBL plasmids flow among humans, livestock, wastewater; routes are poorly mapped.	Which reservoirs drive human acquisition? Can plasmid phylogeography be used in surveillance?
Lineage-specific evolutionary patterns	Major clones show different resistance trajectories; determinants of their success remain unclear.	Which genomic/ecologic traits explain high-risk lineage success under FQ pressure?
Lack of clinical validation for efflux or plasmid-transfer inhibitors	Inhibitors show promising in vitro effects but lack clinical trials.	Which inhibitor classes are most promising clinically? How to design combination therapy trials?
Limited clinical application of AI-based prediction	AI models exist but are not integrated into stewardship.	How accurately can AI predict resistance evolution? How to integrate into clinical workflows?

## 8 Clinical consequences and therapeutic challenges

### 8.1 Impact on empirical therapy

Rising FQ nonsusceptibility continues to erode the reliability of traditional empiric strategies for common Gram-negative infections. Because efficacy and resistance suppression depend on achieving ciprofloxacin AUC/MIC ≥125 (approximately fAUC/MIC ≥90), underexposure is a recurring problem. Clinical audits demonstrate that routine dosing often misses these targets once MICs approach contemporary values, and the risk of failure increases when therapy is initiated without explicit consideration of local MIC distributions or patient-specific PK factors such as renal clearance and body size (De Vroom et al., 2020; Labreche and Frei, 2012; Gai et al., 2019). This challenge is most evident among ESKAPE pathogens, where baseline MIC distributions are frequently shifted upward, narrowing the margin between effective exposure and resistance selection during empiric therapy.

The MSW framework adds a mechanistic explanation. Drug concentrations between the MIC and the MPC favor first-step QRDR mutants and PMQR-primed subpopulations. This argues for early, target-attaining exposure followed by prompt de-escalation when cultures become available, or for selecting a non-FQ agent when PK/PD analysis indicates that suppressive exposure is improbable (Drlica, 2003; Firsov et al., 2003). Failure to recognize this window during empiric therapy creates conditions that actively promote resistance amplification rather than bacterial eradication.

**Figure 6:**
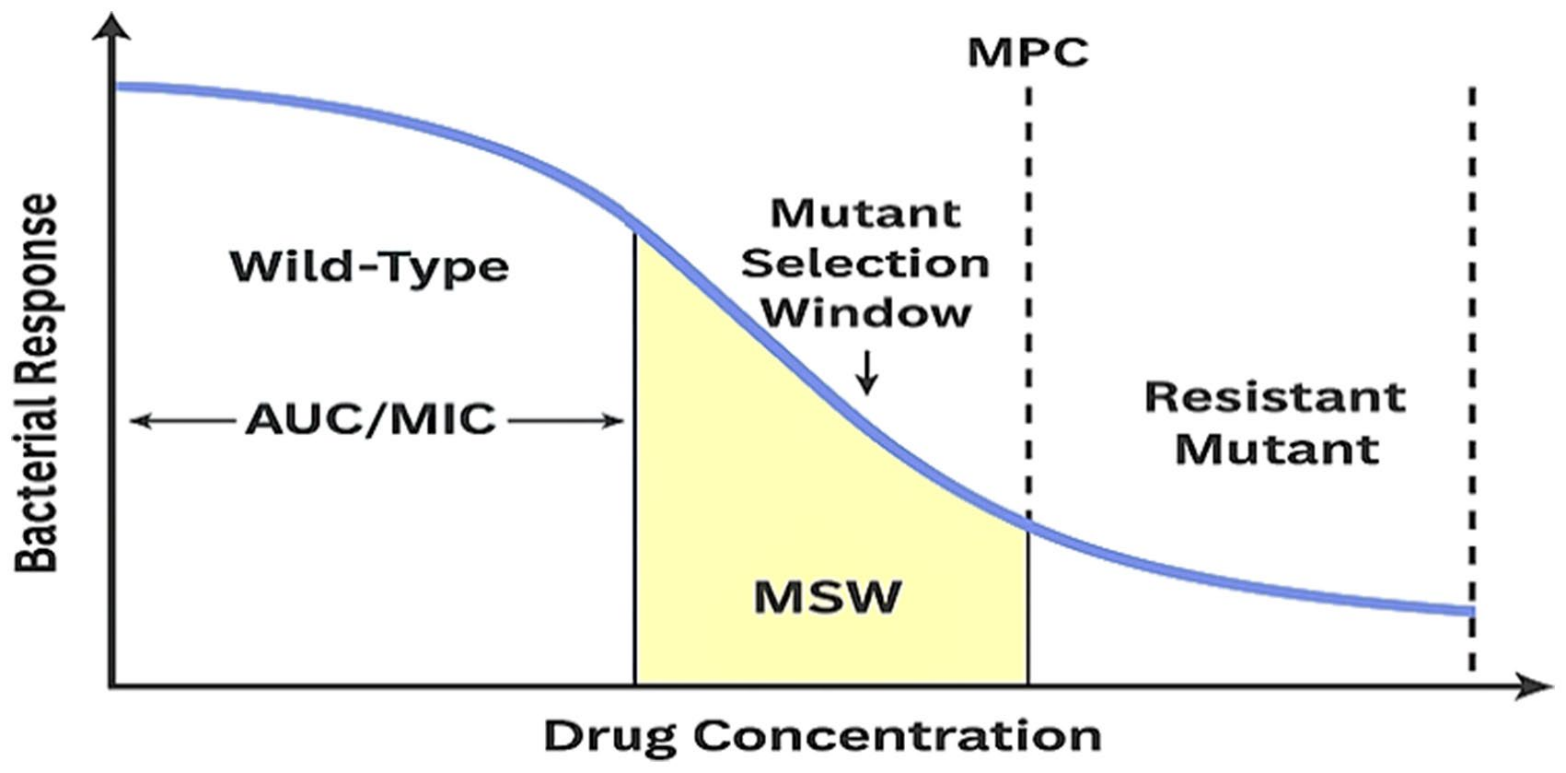
PK/PD model of FQ exposure and resistance selection. Schematic showing the relationship between AMC over time, the MIC, the MPC, and the MSW. The AUC is shown relative to MIC and MPC thresholds. Sustained exposure above MPC suppresses resistance, whereas concentrations within the MSW selectively enrich first-step QRDR mutants and PMQR-primed subpopulations.

Figure 6 summarizes this PK/PD architecture by relating the concentration–time profile to MIC, MPC, and the MSW. Standard clinical regimens frequently allow concentrations to remain within this window, especially as MICs drift upward. This increases the likelihood that low-level resistant subpopulations will expand before therapy is modified.

Clinical outcome data reinforce these PK/PD concerns. Even small increases in MIC within the “susceptible” range lower the probability of reaching an AUC/MIC ≥125 and are consistently associated with reduced clinical and microbiological response. Population PK and probability of target attainment (PTA) analyses show that conventional intravenous ciprofloxacin regimens rarely achieve optimal exposure once MICs reach 0.25–0.5 mg/L, resulting in extended time inside the MSW (Haeseker et al., 2013; Abdulla et al., 2020). Observational studies in Enterobacterales bloodstream and urinary infections link higher MICs with decreased cure and greater treatment failure when exposure is not individualized (Zelenitsky and Ariano, 2010; Fleischner et al., 2020). In *P. aeruginosa*, multiple cohorts associate higher ciprofloxacin MICs with inferior outcomes, including increased mortality, and emphasize the poor PTA achieved against nonfermenters when MICs exceed 0.5 mg/L (Horcajada et al., 2019; Hartman et al., 2020). These effects are magnified in settings where ESKAPE pathogens dominate empirical therapy decisions, as delayed recognition of marginal susceptibility increases both the risk of early clinical failure and the likelihood of resistance amplification.

These data support a stewardship strategy that prioritizes MIC-stratified dosing, Bayesian AUC guidance where available, rapid molecular screening for PMQR and efflux markers, and early movement to alternative agents when suppressive exposure is unlikely. Table 7 summarizes pathogen-specific FQ MIC trends, recommended AUC/MIC targets, and PTA estimates at clinically relevant MIC values across major ESKAPE pathogens, highlighting where rising MICs impede target attainment and elevate the risk of clinical failure.

**Table 7:** Pathogen-specific MIC/PKPD and clinical outcome summary.

Pathogen	MIC shift/resistance trend (last decade)	AUC/MIC target (ciprofloxacin)	PK/PD failure/PTA evidence (key MICs)	References
*E. coli*	ECDC/EARS-Net 2014–2024: rising non-susceptibility in several countries; EU/EEA population-weighted estimates ~15% (2024) with marked country heterogeneity	AUC₀-₂₄/MIC ≥ 125 (commonly cited); some analyses support higher targets (≥250) for severe infections	Population PK/PTA studies show substantial loss of PTA as MIC approaches 0.25–0.5 mg/L; hospitalized cohorts required higher doses to reach AUC/MIC ≥125	Haeseker et al. (2013), Abdulla et al. (2020), European Centre for Disease Prevention and Control ECDC (2025), and Zelenitsky and Ariano (2010)
*K. pneumoniae*	ECDC 2024 documents country-level hotspots with high FQ resistance (>50% in some settings); overall heterogeneity across EU/EEA	AUC₀-₂₄/MIC ≥ 125 (Gram-negative target); consider higher targets for severe infections	PTA modeling indicates rapid decline in target attainment at MIC ≥0.25–0.5 mg/L without dose optimization; clinical failures reported in high-MIC settings	Haeseker et al. (2013) and European Centre for Disease Prevention and Control ECDC (2025)
*P. aeruginosa*	Global surveillance (SENTRY, regional reports) show upward MIC shifts and increased MDR/XDR proportions in many regions over the past decade.	AUC₀-₂₄/MIC ≥ 125 often used, but PTA targets may be unattainable for non-fermenters at higher MICs; Cmax/MIC also relevant.	Monte-Carlo/PTA analyses show very low PTA at MIC ≥0.5–1 mg/L with standard dosing; cohort studies associate elevated MICs (≥0.5 mg/L) with worse clinical outcomes.	Van et al. (2019), Diekema et al. (2019), and Karruli et al. (2023)
*A. baumannii*	Surveillance reports document persistently high FQ non-susceptibility in many regions; *A. baumannii* often exhibits high MIC distributions.	AUC₀-₂₄/MIC ≥ 125 is the general Gram-negative benchmark, but failure common when MICs elevated; alternative agents often required.	PTA and clinical experience indicate poor target attainment at MIC >0.25–0.5 mg/L; high failure rates reported in hospital cohorts.	Abdulla et al. (2020) and Borcan et al. (2025)
*S. aureus*	FQ-R especially in MRSA has risen due to accumulating *gyrA* and *grlA*/*parC* QRDR mutations and efflux pump (NorA) upregulation	For Gram-positive organisms, AUC/MIC >33–50 is associated with effective suppression of mutant selection	Suboptimal exposure within the MSW rapidly enriches first-step *grlA*/*gyrA* mutants, increasing MICs and driving resistance development	Brdová et al. (2024), Li et al. (2016), and Oonishi et al. (2007)

### 8.2 Organ- and syndrome-specific clinical implications of FQ MIC creep

Rising FQ MICs have predictable consequences across clinical syndromes because drug exposure and PK/PD target attainment differ markedly between organ systems (Roger, 2024). As a result, an MIC that is acceptable at one site of infection may be inadequate at another, complicating empiric treatment decisions (de Vroom et al., 2020). In complicated urinary tract infections, ciprofloxacin normally achieves high urinary and renal concentrations, yet population PK/PD analyses show that the probability of reaching the recommended AUC/MIC target declines sharply once MICs approach 0.25–0.5 mg·L^−1^. Clinical series link these borderline MICs to higher microbiologic relapse and increased clinical failure, even when doses are adjusted for renal function (Haeseker et al., 2013; Jakobsen et al., 2020; Seok et al., 2018).

In hospital-acquired and VAP, attainment depends on epithelial lining fluid exposure. Multiple PK/PD and cohort studies demonstrate that *P. aeruginosa* isolates with ciprofloxacin MIC ≥0.5–1 mg·L^−1^ have markedly reduced probability of target attainment and are associated with greater clinical failure and mortality in ICU populations. Limited pulmonary penetration magnifies the impact of even modest MIC elevation in these settings. Efflux-driven resistance in nonfermenters compounds this problem by elevating MICs and lowering achievable intracellular and epithelial lining fluid concentrations (Van et al., 2019; Ramírez-Estrada et al., 2016).

For bloodstream infections, observational cohorts and systematic analyses show that incremental MIC increases still within older susceptible breakpoints correlate with worse outcomes. Several studies report reduced clinical cure and higher mortality as ciprofloxacin MICs rise toward 0.25–0.5 mg·L^−1^, consistent with widespread AUC/MIC non-attainment in hospitalized adults (Falagas et al., 2012; Tumbarello et al., 2007). Here, limited dosing flexibility and rapid bacterial replication further narrow the therapeutic margin.

In skin and soft-tissue infections (SSTI) caused by *S. aureus*, especially strains with GrlA/ParC substitutions and Nor pump overexpression, elevated MICs reduce intracellular and tissue exposure. These changes contribute to slower clinical responses and higher recurrence rates when FQs are used empirically, particularly in settings where methicillin-resistant strains predominate (Jakobsen et al., 2020; Robicsek et al., 2006b).

These clinical data support several practical stewardship principles: avoid FQs for invasive infections when MICs approach epidemiologic breakpoints; consider PK-guided optimization or alternative therapy in complicated urinary infections with borderline MICs (~0.25–0.5 mg·L^−1^); and limit empirical use in settings with evident MIC drift or high prevalence of resistant *S. aureus*. These recommendations reflect convergent PK/PD evidence showing frequent AUC/MIC non-attainment at these MIC values and clinical cohort findings that consistently associate MIC rise with increased risk of treatment failure (Haeseker et al., 2013; Jakobsen et al., 2020; Falagas et al., 2012).

### 8.3 Stewardship and surveillance decision algorithm

Translating PK/PD principles into day-to-day practice requires a simple framework that incorporates MIC values, early molecular markers of reduced susceptibility, and basic indicators of efflux or permeability involvement. Clinical PK/PD audits consistently show that standard ciprofloxacin regimens often fail to reach recommended AUC/MIC targets in hospitalized patients, leaving concentrations within the mutant selection window and increasing the likelihood of selecting first-step QRDR mutants (Haeseker et al., 2013). Molecular surveys demonstrate that low-level PMQR determinants (*qnr, aac(6′)-Ib-cr, qepA, oqxAB*) frequently accompany early QRDR substitutions and facilitate rapid amplification under marginal drug exposure, which supports the use of genotype-informed thresholds when making treatment decisions (Kotb et al., 2019). These findings highlight that dependence on phenotypic susceptibility testing alone can overlook early resistance evolution during empirical therapy, particularly when low-level or emerging mechanisms are present (Urmi et al., 2020).

Because MPC and MSW estimates vary by strain and assay conditions, the algorithm emphasizes confirmatory phenotyping or model-informed (Bayesian) AUC estimation for borderline cases, and alignment of decision thresholds with local surveillance data from GLASS and national AMU indicators (World Health Organization, 2022b; Krajewska et al., 2023; Alnezary et al., 2023). This approach allows stewardship decisions to account for both exposure adequacy and the underlying genetic context of reduced susceptibility. In practice, isolates with MIC values close to the breakpoint combined with PMQR or efflux signatures should trigger avoidance of FQs, initiation of targeted genomic surveillance, and selection of alternative agents. Borderline MICs without PMQR may be considered for PK optimization through dose adjustment or Bayesian AUC estimation, with close clinical and microbiologic follow-up. Isolates with clearly low MICs and no PMQR markers can be managed with standard stewardship procedures and routine monitoring (World Health Organization (WHO), 2024).

**Figure 7:**
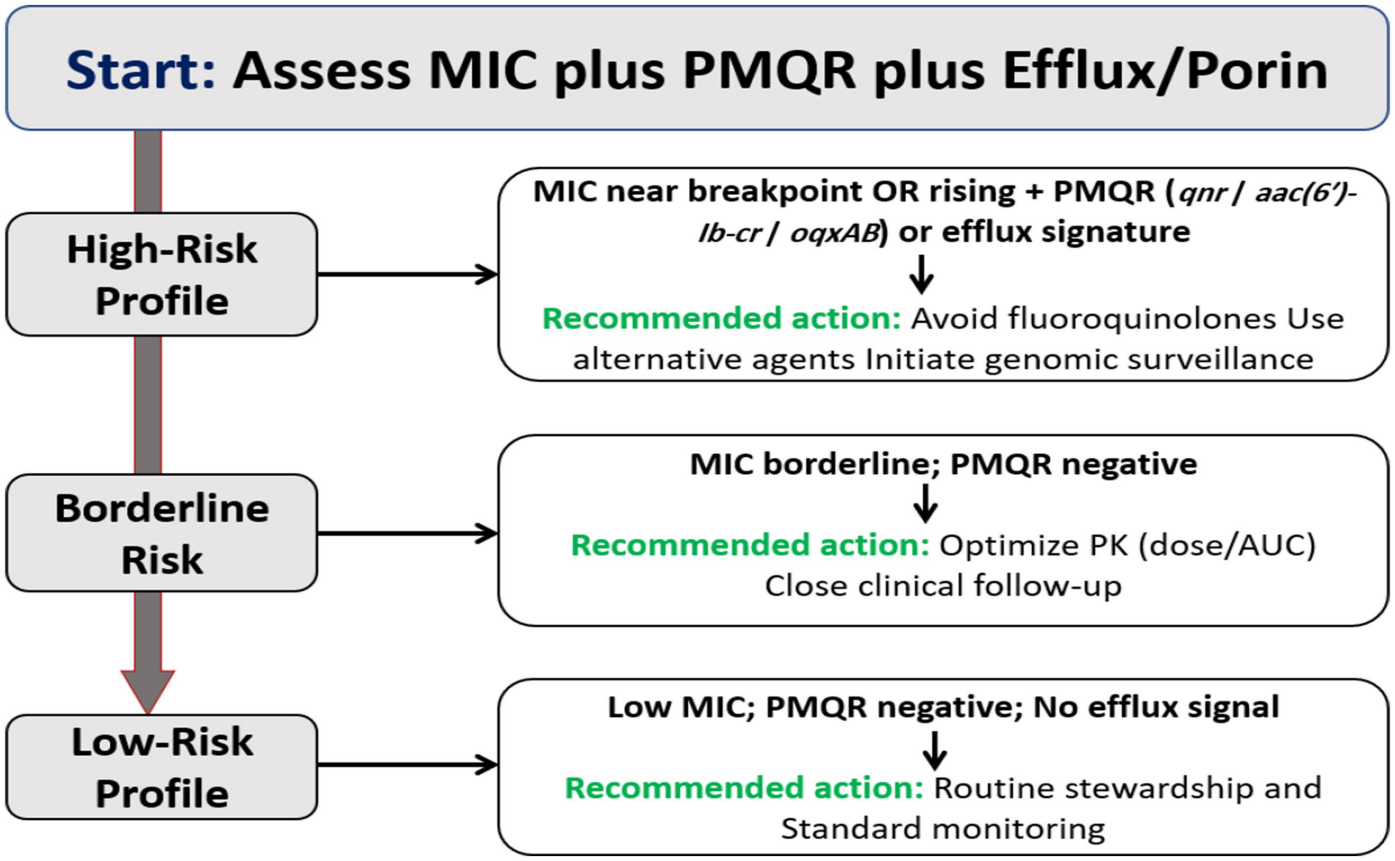
FQ stewardship and surveillance decision algorithm. Algorithm integrating MIC, PMQR determinants (*qnr, aac(6′)-Ib-cr, oqxAB*) and efflux or porin indicators to guide FQ prescribing and surveillance. Isolates are organized into high-, borderline-, and low-risk categories based on MIC position relative to the breakpoint and the presence of PMQR markers or efflux-associated signals. Recommended actions include FQ avoidance with genomic surveillance for high-risk isolates, PK-guided optimization and clinical follow-up for borderline isolates, and routine stewardship measures for low-risk isolates.

Figure 7 provides a pragmatic decision framework based on MIC category, PMQR status, and markers of efflux or porin involvement. It separates isolates into high-, borderline-, and low-risk groups and outlines corresponding actions ranging from avoidance of FQs to optimized dosing or standard management. This structure operationalizes the PK/PD to evolution to clonal expansion model and supports consistent, evidence-based decision making across clinical and surveillance settings. By explicitly linking microbiologic, pharmacologic, and surveillance inputs, this framework supports consistent and reproducible stewardship decisions across clinical and monitoring settings.

### 8.4 Combination strategies and novel adjuvants (efflux inhibitors, OM permeabilizers)

Mechanism-based potentiation seeks to restore intracellular FQ exposure by reducing efflux or improving entry. In *S. aureus*, NorA inhibition decreases FQ MICs and slows resistance development *in vitro*; several compound classes function as efflux-pump inhibitors, although none is suitable as monotherapy (Aeschlimann et al., 1999; Palazzotti et al., 2023; Zimmermann et al., 2019; Martin et al., 2024). These findings support adjuvant rather than standalone use, particularly in settings where QRDR mutations alone do not fully explain elevated MICs. In Gram-negative pathogens, overexpression of RND and MFS systems raises MICs in an additive manner. In Enterobacterales, increased AcrAB-TolC activity and plasmid-encoded *oqxAB* frequently accompany higher FQ MICs, making these pumps reasonable adjuvant targets (Bialek-Davenet et al., 2015; Wand et al., 2022; Razavi et al., 2020). Such combinations are most valuable when modest resistance mechanisms, such as efflux overexpression, coexist with marginal PK/PD exposure, a context in which adjuvants can help prevent progression from low-level to clinically significant resistance (Ebbensgaard et al., 2020).

OM permeabilizers provide a complementary strategy. Polymyxins and related agents can transiently disrupt the outer membrane, increase intracellular drug accumulation, and generate synergy in *P. aeruginosa* and other nonfermenters, an effect linked to altered lipopolysaccharide structure and enhanced uptake (Poole et al., 2015; Delcour, 2009; Wang et al., 2022; Zheng et al., 2020). Because these approaches primarily enhance access rather than alter target affinity, their benefit depends on careful stewardship and resistance mechanism profiling. These findings support combination approaches in which an efflux inhibitor or membrane-active partner increases target access and delays resistance amplification, with use restricted to settings where pharmacologic benefit clearly outweighs toxicity or collateral-selection risks.

### 8.5 Collateral sensitivity and emerging treatment paradigms

Resistance pathways impose trade-offs that may be therapeutically exploitable. MSW-aware dosing, which delivers early target-attaining exposure followed by rapid de-escalation, reduces time within the selective range and limits enrichment of first-step mutants (Drlica, 2003; Drlica and Zhao, 2007). Mechanism-informed sequencing or combination therapy can extend this concept. Pairing a FQ with an agent that capitalizes on efflux-related vulnerabilities or OM alterations may restore activity or slow resistance evolution. Experimental data on NorA-directed adjuvants, modulation of AcrAB and *oqxAB*, and co-administration of OM with active agents outline a coherent rationale for such strategies (Aeschlimann et al., 1999; Bialek-Davenet et al., 2015; Wang et al., 2022).

Recent work shows that models trained on surveillance, clinical, and genomic data can predict FQ susceptibility and MICs with high accuracy. These tools can highlight settings, lineages, and periods with a higher risk of rising MIC values and can support early stewardship or targeted sampling (Kim et al., 2022; Sakagianni et al., 2023; Pataki et al., 2020). Reviews and methodological studies report that different modeling approaches, including k-mer based, gene presence based, and mutation focused methods, predict ciprofloxacin resistance with strong performance across Enterobacterales, *Pseudomonas*, and other major pathogens. These findings support the value of such models in resistance surveillance (Farhat et al., 2023; Gao et al., 2024). When integrated with antimicrobial use data, these approaches provide a population-level tool for anticipating resistance trajectories rather than reacting to established clinical failure.

Several nanoparticle delivery platforms have also progressed. Polymeric carriers, lipid systems, silver-based formulations, and metal organic frameworks improve drug delivery and increase bacterial uptake. Many of these systems also enhance activity against biofilms and strengthen the effect of quinolones in preclinical and ex vivo studies (Yadav et al., 2022; Ibraheem et al., 2022; Negro et al., 2023). Examples include fucoidan coated chitosan nanoparticles and silver ciprofloxacin formulations that reduce MIC values and disrupt biofilms in resistant *Salmonella* and *Pseudomonas*. Other systems such as zirconium based and metal organic frameworks allow controlled drug release and improve intracellular drug levels in infected tissues (Ibraheem et al., 2022; Elbi et al., 2017; Aden et al., 2023). By raising drug concentrations at the site of infection, these delivery systems may compress the MSW and thereby lower the risk of resistance amplification when exposure is otherwise suboptimal (Drlica and Zhao, 2007).

Forecasting tools can help determine where enhanced stewardship or supportive agents are needed, while nanoparticle delivery may narrow the mutant selection window by increasing local drug concentrations and overcoming reduced permeability and efflux. Both approaches require clinical studies and One Health surveillance to assess effectiveness, safety, and ecological impact (Kim et al., 2022; Lucky and Kusi, 2025).

Although clinical validation is incomplete, these approaches align with the broader principle of integrating PK/PD optimization with targeted manipulation of resistance pathways. Table 8 provides a structured overview of PK/PD targets (AUC/MIC and MPC/MSW), efflux- and membrane-focused adjuvants, and collateral-sensitivity combinations to guide cautious, stewardship-aligned use of FQs as part of combination or sequential regimens.

**Table 8:** Therapeutic levers for managing FQ resistance: PK/PD targets, adjuvants, and paradigms.

Lever/Mechanism	Representative examples	Primary context	Therapeutic implication	Evidence type	References
PK/PD target attainment (AUC/MIC ≥125)	Target: AUC/MIC ≥125; integrate patient PK and local MICs	Gram-negative infections (cUTI, intra-abdominal infection, hospital-acquired pneumonia)	Avoid empiric FQs when target attainment unlikely; optimize dose early and de-escalate	K/PD analyses; clinical audits	De Vroom et al. (2020), Labreche and Frei (2012), Gai et al. (2019)
MSW/MPC principle	Front-loaded exposure; rapid de-escalation once cultures return	Broad (Gram-negative & Gram-positive)	Reduces enrichment of first-step QRDR mutants; supports optimized short FQ courses	PK/PD theory; in vitro selection	Drlica (2003), Drlica and Zhao (2007), and Firsov et al. (2003)
Efflux inhibition (NorA)	NorA EPIs (repurposed and novel scaffolds)	*S. aureus*	Lowers FQ MICs; delays resistance, adjuvant use	In vitro; mechanistic	Aeschlimann et al. (1999), Palazzotti et al. (2023), Zimmermann et al. (2019), and Martin et al. (2024)
Efflux modulation (AcrAB-TolC / OqxAB)	Targeting AcrAB-TolC, *oqxAB* overexpression	Enterobacterales	Adjuvant target to restore intracellular FQ exposure; pairs with β-lactams/OM-active agents	Genomics; phenotype–genotype studies	Bialek-Davenet et al. (2015), Wand et al. (2022), and Razavi et al. (2020)
Outer membrane permeabilization	Polymyxins; newer membrane-active compounds	*P. aeruginosa* and other Gram-negative	Increasing of Intracellular FQ exposure; synergy possible under stewardship controls	Mechanistic reviews; in vitro/PK	Zeng et al. (2024), Poole et al. (2015), Delcour (2009), and Wang et al. (2022)
Combination therapy (mechanism-guided)	FQ + β-lactam ± EPI/OM partner	Gram negative & selected Gram-positive	Slows resistance amplification; exploits collateral vulnerabilities	Mechanistic rationale; preclinical; limited clinical	Labreche and Frei (2012), Poole et al. (2015), Delcour (2009), and Wang et al. (2022)
Collateral sensitivity-informed sequencing	Cycle to drug/class for which resistant state is hypersusceptible	Organism−/mechanism-specific	Framework for sequential therapy to delay resistance; needs local data	Conceptual/experimental	Drlica (2003) and Drlica and Zhao (2007)

### 8.6 Emerging FQs and next-generation topoisomerase inhibitors

Several newer FQs and non-quinolone type II topoisomerase inhibitors are now in clinical use or late-stage development, and they require surveillance that links molecular mechanisms with evolving resistance patterns. This challenge is amplified in empiric therapy, where treatment decisions are frequently made before organism-specific MIC data are available, increasing reliance on population-level susceptibility patterns.

For the newer FQs (for example delafloxacin, levonadifloxacin/alalevonadifloxacin, sitafloxacin), resistance generally follows the same molecular routes that shape classical FQ-R. Clinical and laboratory studies repeatedly show that reduced susceptibility is driven by QRDR substitutions in *gyrA* and *parC* together with increased efflux, even though each agent displays distinct potency and species-specific activity profiles (Turban et al., 2023; Douglas and Laabei, 2023). Delafloxacin maintains activity in acidic environments and retains activity against some resistant *S. aureus* and Gram-negative isolates, but decreased susceptibility has been associated with accumulated *gyrA* and *parC* substitutions and efflux phenotypes (Seok et al., 2018; Ramírez-Estrada et al., 2016). Sitafloxacin retains potency against many ESBL-producing Enterobacterales, yet elevated MICs in clinical isolates have been linked to multilocus QRDR mutations frequently accompanied by plasmid-mediated determinants such as *qnr* or *aac(6′)-Ib-cr*, or by enhanced efflux (Rezaei et al., 2024; Zhang et al., 2025b).

By contrast, several next-generation non-quinolone topoisomerase inhibitors such as the spiropyrimidinetriones (for example, zoliflodacin) bind distinct regions of GyrB or ParE and select for different target-site substitutions. Early surveillance and laboratory evolution studies indicate limited cross-resistance with classical quinolones, although efflux systems can still diminish susceptibility; for instance, MtrCDE in *Neisseria gonorrhoeae* reduces activity of these compounds despite their structural divergence (Jacobsson et al., 2022; Le et al., 2021; Collins et al., 2024).

These findings suggest that new FQs will continue to follow the canonical QRDR- and efflux-based resistance trajectories, whereas non-quinolone type II inhibitors may temporarily avoid classical QRDR cross-resistance but remain vulnerable to efflux and to newly emerging target mutations. This underscores the need for prospective genotypic and phenotypic surveillance to detect early shifts in resistance as these agents are adopted clinically (Jacobsson et al., 2019; Mlynarczyk-Bonikowska et al., 2022).

## 9 Future directions

Slowing fluoroquinolone resistance will require coordinated strategies that integrate molecular mechanisms, PK/PD principles, and One Health surveillance. Recent advances provide an opportunity to strengthen this approach, particularly for ESKAPE pathogens, which account for a disproportionate share of AMR burden and clinical impact across healthcare and connected environments (Miller and Arias, 2024). Predictive models that analyze genomic, phenotypic, and AMU data show strong performance in estimating resistance patterns and MICs in *E. coli* and other Gram-negative pathogens. These tools may help identify high-risk clones and settings before resistance becomes clinically evident, thereby informing earlier stewardship interventions (Kim et al., 2022; Sakagianni et al., 2023; Pataki et al., 2020).

Several nanoparticle systems have also advanced. Chitosan carriers, silver ciprofloxacin complexes, and metal–organic frameworks improve intracellular drug delivery and biofilm penetration in preclinical studies. Many of these platforms also enhance ciprofloxacin activity against resistant Enterobacterales and non-fermenters (Ibraheem et al., 2022; Elbi et al., 2017; Aden et al., 2023). These approaches may help overcome reduced permeability and efflux and could complement optimized dosing strategies. Their future use, however, depends on clinical evaluation, standardized production methods, safety assessment, and coordinated environmental monitoring. Their integration into clinical practice will therefore need to align with stewardship goals and include assessment of ecological impact across human, animal, and environmental compartments, consistent with a One Health approach (World Health Organization, 2015).

Three priorities consistently shape future work: predictive tools, inhibition of resistance drivers, and translation of biology-focused therapies. Computational models now link genomic and drug-use patterns to likely resistance outcomes. They can also highlight mutational paths and exposure regimens that steer bacterial populations toward less fit or less transmissible states. Their utility will depend on validation in real clinical settings and consistent data sharing, including harmonization with national and international AMR surveillance programs (Pinheiro, 2024; Branda and Scarpa, 2024; Wang et al., 2025).

Efflux activity and plasmid transmission remain major barriers. New efflux inhibitors show improved selectivity, with NorA being the leading target in Gram-positive species and AcrAB-TolC and OqxAB being central in Gram-negative organisms. When combined with optimized drug exposure or beta lactams, these agents can increase intracellular FQ levels and reduce selection of resistant subpopulations. Blocking plasmid transfer through inhibition of relaxase and related proteins may also limit spread of plasmid-mediated determinants, an approach with potential relevance across clinical, veterinary, and environmental reservoirs (Duffey et al., 2024; Zhang et al., 2024; Alenazy, 2022; Williams et al., 2025).

Therapies directed at specific biological structures provide additional options. Bacteriophages, including variants designed to improve penetration of bacterial capsules and biofilms, have shown synergy with FQs in experimental models, and early clinical reports suggest acceptable safety profiles. Nanoparticle formulations, including metallic, lipid, polymeric, and hybrid platforms, improve targeted delivery, increase uptake, and enhance oxidative damage in bacterial cells. Their development will require careful dose selection, safety studies, and integration with strategies that minimize selection of new resistance patterns, particularly in settings with high antimicrobial exposure pressure (Sawa et al., 2024; Uchechukwu and Shonekan, 2024; Nir-Paz et al., 2025; Peng et al., 2024; Rodrigues et al., 2024; Mondal et al., 2024; Parvin et al., 2025; Marfavi et al., 2024).

Rapid diagnostics reinforce these efforts. Genotypic panels, targeted sequencing, and improved culture workflows shorten time to appropriate therapy when used with diagnostic stewardship and real-time clinical support. Including plasmid and mutation data relevant to QRDR and PMQR markers can support mechanism-guided treatment decisions, earlier de-escalation, and selection of suitable adjuvant combinations (Peri et al., 2024; Moore et al., 2023). Overall, predictive modeling, efflux and plasmid inhibition, targeted bacteriophage and nanoparticle therapies, and rapid diagnostics provide a coherent path toward slowing FQ-R while maintaining clinical effectiveness, consistent with One Health priorities for sustainable AMU.

## 10 Conclusion

FQ-R continues to expand through species-specific evolutionary routes shaped by antimicrobial exposure, clonal background, and cross-sector transmission. Across the ESKAPE pathogens, resistance is rooted in QRDR substitutions and reinforced by plasmid-mediated determinants such as *qnr*, *aac*(6′)-*Ib-cr*, *qepA*, and *oqxAB*, together with organism-specific efflux and permeability pathways. These shared molecular foundations explain the convergence of resistance trajectories across diverse infections and ecological settings despite pathogen-level variation.

These mechanisms interact with PK and PD constraints, particularly suboptimal AUC/MIC target attainment and prolonged residence within the MSW, which favor first-step mutants and support the expansion of high-risk lineages including *E. coli* ST131, *K. pneumoniae* CG258, *A. baumannii* IC2, and *P. aeruginosa* ST235. As MIC distributions shift upward, the likelihood of achieving suppressive exposure with standard dosing declines, narrowing the margin in which empirical fluoroquinolone therapy remains reliable.

One Health evidence indicates that comparable selection pressures operate beyond clinical settings. Veterinary quinolone use promotes enrichment of PMQR determinants in food animals, wastewater, and natural waters, facilitated by mobile plasmids such as IncX, IncF, and IncL. These reservoirs enable continued reintroduction of resistance determinants into human populations and limit the effectiveness of hospital-focused containment strategies alone. This reinforces the importance of integrated surveillance frameworks that combine GLASS-AMR, GLASS-AMC, the WHO Tricycle protocol, and environmental indicators.

Clinically, rising MICs increasingly constrain FQ use, particularly in pneumonia and bacteremia, where achieving effective exposure is difficult. Stewardship therefore depends on early exposure optimization, timely de-escalation, and avoidance of FQs when MICs, resistance markers, or clinical context predict poor target attainment. Incorporating MIC-aware prescribing and early resistance signals into empirical decision making is now essential.

Continued progress will require focused attention to efflux and plasmid-transfer biology, development of targeted adjuvants, refinement of bacteriophage and nanoparticle approaches, and broader implementation of rapid, mechanism-aligned diagnostics. Aligning these strategies with PK/PD principles and One Health surveillance remains central to preserving the clinical utility of FQs.
